# Evolution of p53 Transactivation Specificity through the Lens of a Yeast-Based Functional Assay

**DOI:** 10.1371/journal.pone.0116177

**Published:** 2015-02-10

**Authors:** Mattia Lion, Ivan Raimondi, Stefano Donati, Olivier Jousson, Yari Ciribilli, Alberto Inga

**Affiliations:** 1 Laboratory of Transcriptional Networks, Centre for Integrative Biology (CIBIO), University of Trento, Mattarello, Trento, Italy; 2 Laboratory of Microbial Genomics, Centre for Integrative Biology (CIBIO), University of Trento, Mattarello, Trento, Italy; University of Saarland Medical School, GERMANY

## Abstract

Co-evolution of transcription factors (TFs) with their respective *cis*-regulatory network enhances functional diversity in the course of evolution. We present a new approach to investigate transactivation capacity of sequence-specific TFs in evolutionary studies. *Saccharomyces cerevisiae* was used as an *in vivo* test tube and p53 proteins derived from human and five commonly used animal models were chosen as proof of concept. p53 is a highly conserved master regulator of environmental stress responses. Previous reports indicated conserved p53 DNA binding specificity *in vitro*, even for evolutionary distant species. We used isogenic yeast strains where p53-dependent transactivation was measured towards chromosomally integrated p53 response elements (REs). Ten REs were chosen to sample a wide range of DNA binding affinity and transactivation capacity for human p53 and proteins were expressed at two levels using an inducible expression system. We showed that the assay is amenable to study thermo-sensitivity of frog p53, and that chimeric constructs containing an ectopic transactivation domain could be rapidly developed to enhance the activity of proteins, such as fruit fly p53, that are poorly effective in engaging the yeast transcriptional machinery. Changes in the profile of relative transactivation towards the ten REs were measured for each p53 protein and compared to the profile obtained with human p53. These results, which are largely independent from relative p53 protein levels, revealed widespread evolutionary divergence of p53 transactivation specificity, even between human and mouse p53. Fruit fly and human p53 exhibited the largest discrimination among REs while zebrafish p53 was the least selective.

## Introduction

*cis*-regulatory elements (CREs) are defined as regions of DNA containing a set of transcription factor binding sites (or response elements, REs) recognized by sequence-specific transcription factors (TFs). Latest evidences are highlighting that divergence in *cis*-regulatory sequences might underlie phenotypic diversity in evolution more commonly than divergence in the sequences of the TFs themselves. Gain, loss or changes in the affinity of TFs at the level of REs can indeed shape gene regulatory networks [1] [2]. In addition, co-evolution of *cis*-regulatory elements and of TF proteins is emerging as an extremely important evolutionary process, defined as *cis-trans* co-evolution [3].

Many important TFs, especially those involved in development, or acting as master regulators, appeared to be highly conserved at the functional level during evolution, as revealed by ectopic expression of orthologous genes and complementation assays [2] [3] [4].

Among those TFs, the tumor suppressor protein p53 emerges as one clear example of this functional conservation. p53 is one of the most studied sequence-specific TF and its inactivation frequently occurs in cancer through the expression of mutant p53 proteins [5] [6] [7]. p53 plays a crucial role in the control of genome stability, apoptosis, cell cycle, cellular senescence and angiogenesis; for these reasons it is known as ‘‘the guardian of the genome” [6]. Being a master regulator, p53 can also influence many other biological processes including autophagy, mTOR signaling, cell motility and migration, energy and amino acid metabolism, immune response, to name just a few [8] [9] [6] [7].

To modulate so many different biological responses and to control the expression of a myriad of genes, p53 has to be tightly regulated [8] [10]. p53 specifically recognizes a consensus sequence composed of two decamers that can be separated by a short spacer (5’-RRRCWWGYYY-N-RRRCWWGYYY-3’; R = purine, Y = pyrimidine and W = adenine or thymine; N = spacer) [11] [12]. p53 binds the RE as a tetramer (dimer of dimers) and differences in RE sequences can strongly impact its transactivation capacity [13]. Many studies have investigated the role of RE sequence in transactivation potential and specificity of human p53 protein [14] [13] [12], revealing the crucial role of the core CWWG in determining the level of transactivation, with CATG leading to highest activity followed by CAAG and CTTG sequences and by CTAG associated with the weakest responsiveness. The flanking purines and pyrimidines are also very important, and, in particular, the dinucleotide motifs flanking the core lead to highest transactivation when their sequence is GG or CC and lowest when the sequence is AG or CT [13]. Interestingly, most p53 REs deviates from the optimal consensus and low-affinity REs appear to have been selected to fine-tune the activation of specific p53-induced pathways, such as apoptosis [14] [15] [13] [16]. Non-canonical p53-REs have also been annotated, consisting of 1/2- (a decamer) and 3/4-sites (a decamer + 1/4-site) [17] [18] [16].

These findings have implications when animal models are employed for the study of p53 or of transcription factors in general, as RE differences occur even between closely related species. For instance, while human and mouse p53 proteins share high sequence identity [19] and exhibit highly overlapping structure [20] as well as similar biochemical properties and transactivation potential, at least towards high affinity REs [21], evolutionary divergence is more evident at the level of p53 REs. Indeed, several genes involved in DNA metabolism and repair that are p53 targets in humans are not responsive to p53 in rodents, which could explain the differences observed in response to environmental stress, cellular damage, and cancer development [19] [22] [23]. Sources of evolutionary diversity in p53 regulatory networks between mammalian species have been identified in intronic fuzzy tandem repeats in mouse [24] and endogenous retroviruses (ERVs) in humans [25] containing p53 REs.

Here we investigated changes in transactivation specificity for p53 proteins derived from *Homo sapiens* and the animal models *Mus musculus*, *Xenopus laevis*, *Danio rerio*, *Drosophila melanogaster*, and *Caenorhabditis elegans*. We focused on the impact of RE sequence, p53 protein level and temperature on transactivation using a yeast-based functional assay that minimizes the impact of variables such as chromatin state, promoter landscape and the influence of cofactors.

## Materials and Methods

### Yeast strains and culture media

Ten isogenic yeast strains (yLFM), auxotrophic for tryptophan and containing different human p53 response element (RE) sequences cloned upstream the reporter Firefly luciferase gene were tested [26] [27]. The panel of reporter strains comprises five human REs from downstream target genes of p53 (p21, MDM2 P2, MMP2, BAI1, and PUMA) and five *ad hoc* generated variations of the p53 consensus sequences (canonical CON1, CON2, CON3 and 3/4-sites, CON-J and CON-K [16]). Three additional reporter strains were constructed starting from the previously described yLFM-ICORE strain [23] and a targeting oligonucleotide containing the sequence of the potential p53 RE from the *C*. *elegans* ced-13 target gene [28] or the two putative p53 REs from the *X*. *laevis* p21 and mdm2 target gene (S1 File). Strain construction was performed and verified as previously described [23].

Rich medium {YPDA; 1% yeast extract, 2% peptone, 2% dextrose (D-glucose), 200 mg/L adenine} was used to expand yLFM strains. Yeast cells were transformed with the appropriate pTSG-based plasmid using the lithium acetate transformation protocol [29] and selected in medium containing dextrose (2%) as carbon source and adenine (200 mg/L) but lacking tryptophan—SDtA- {0.67% Yeast nitrogen base w/o amino acids (Difco, BD Bioscience, Milan, Italy), 50ml/L of non-essential amino acids (αα), 200 mg/L adenine, 1% leucine, 1% histidine, 1% uracil, 1% lysine} [30]. For bacterial cells Luria-Bertani broth (LB, Sigma, Milan, Italy) medium supplemented with ampicillin antibiotic was used for plasmid selection.

Development of Xl_p53 and Dm_p53 expression plasmids in yeast.

pTSG plasmid [30] is based on the centromeric pRS314 plasmid [31] and contains the TRP1 selection marker, the finely-tuned inducible *GAL1* promoter and the ampicillin resistance (ampR) gene for selection in bacterial cells [27].

pTSG-Hs_p53 (*Homo sapiens*) [27], pTSG-Mm_p53 (*Mus musculus*) [23], pTSG-Dr_p53 (*Danio rerio*), pTSG-Cep1 (*Caenorhabditis elegans*), were already available [32]. *Xenopus laevis* cDNA sources were kindly provided by Simona Casarosa’s group in Trento, Italy (CIBIO). pCS2-Dm_p53 plasmid containing the *Drosophila melanogaster* p53 open reading frame (ORF) was kindly provided by Lucio Collavin’s group in Trieste, Italy (L.N.CIB).

The ORF of both *Xenopus laevis* (Xl_p53) and *Drosophila melanogaster* p53 (Dm_p53) were amplified using the Q5 Hot Start High-Fidelity DNA Polymerase (New England Biolabs, Euroclone, Milan, Italy). Primers were designed to amplify the entire p53-ORF of interest and to harbor flanking regions that recombine with the homologous sequences in the multi-cloning site of the pTSG vector (Eurofins MWG Operon, Ebersberg, Germany; sequences available upon request). The p53 ORFs of interest were specifically inserted in the multiple cloning site (MCS) downstream the *GAL1* promoter and upstream of transcription terminator derived from the *CYC1* gene. The recombination process occurs *in vivo* in yeast cells through a gap repair transformation assay [33] using pTSG plasmids digested with BamHI HF and XhoI restriction enzymes (New England Biolabs). In yeast, the linear plasmid is resealed together with the PCR products by the recombination system exploiting the sequence homology at the end of the fragments.

After the *in vivo* cloning, plasmid DNA was recovered from yeast transformants, transformed into *Escherichia coli* competent cells (*XL-1 Blue*) using the Potassium-Calcium-Magnesium method (bakerlab/Cells.htm), extracted from *E*.*coli* transformants (QIAprep Spin Miniprep Kit, QIAGEN), verified by DNA sequencing (BMR Genomics, Padua, Italy) and used in the yeast functional assay. All (4/4) the cDNA collected from *Xenopus laevis* presented the Tyr179Ser sequence variant, which may represent a natural polymorphism. We cannot formally exclude that this variant can have some impact on Xl_p53 transactivation specificity or temperature sensitivity.

### Development of chimeric Dm_p53, Cep-1, and Ta_p53 expression plasmids in yeast

Two different types of chimeras were generated. In the type-one chimera, a portion of the human N-terminal region (1–63 aa, *hN*_*63*_, corresponding to the presence of both TADs) was fused at the 5’ of the full-length sequence of Dm_p53 or Cep-1. The type-two of chimera was instead generated replacing the predicted corresponding N-terminal regions of Dm_p53 or Cep-1 with the entire human N-terminal portion, 1–92 aa hN_92_. The two regions corresponding to hN_63_ and hN_92_ were amplified by PCR from the pTSG-Hs_p53, using the Q5 Hot Start High-Fidelity DNA Polymerase (New England Biolabs). Primers were designed to amplify the Hs_p53 portions of interest and to obtain amplicons that recombine at 5’ with the homologous sequences in the multicloning site of the pTSG vector and at the 3’ with the homologous sequences within Dm_p53 or Cep-1 ORF (Eurofins MWG Operon; sequences available upon request). The recombination occurs *in vivo* in yeast cells through a Gap repair transformation assay using pTSG plasmids single digested with BamHI HF restriction enzyme (New England Biolabs). The amplicons of interest were specifically inserted downstream the *GAL1* promoter and, either upstream the Dm_p53 or Cep-1 ORF, or replaced to their corresponding N-terminal region. The resulting type-one chimera plasmids were named as pTSG-hN_63-_Dm_p53 and pTSG-hN_63-_Cep-1. Alternatively, the resulting type-two chimera plasmids were named as pTSG-hN_92-_ΔNDm_p53, pTSG-hN_92-_ΔNCep-1_A_, pTSG-hN_92-_ΔNCep-1_B_ and pTSG-hN_92-_ΔNCep-1_C_. According to our alignment analysis and previous reports [34] [35] [36], the predicted N-terminal region of Dm_p53 encompasses residues 1–73. We identified three putative N-terminal portions for Cep-1: (A) predicted from our analysis, residues 1–164; predicted from previous study: residues 1–205 (B) [35]; residues 1–220 (C) [37]. After the *in vivo* cloning, plasmid DNA was recovered from yeast transformants and processed as described above.

### Luciferase quantitative assay

yLFM yeast strains were transformed with the appropriate pTSG plasmid or the empty vector pRS314 (as vehicle control) and selected for 48 h at 30°C on SDtA plates [30]. Single colonies were then patched on SDtA plates and grown for additional two days at 30°C. Cells from patches were inoculated on a 96-well plate in 60 μl of media containing 2% raffinose (Fluka, Sigma-Aldrich, Milan, Italy)-SRtA- as carbon source or 2% raffinose supplemented with two different galactose (Sigma-Aldrich) concentrations (0.008% and 0.064%) to induce the *GAL1* promoter and modulate p53 protein levels. Small-volume format yeast-based luciferase assay was performed after vigorous shaking for 6 hrs at three different temperatures: 24°C, 30°C, 37°C. For every transformant four different biological samples (corresponding to four patches) were tested.

Following the 6hrs-incubation, 10 μL of cells were transferred on a white 384-well plate and incubated in agitation for 15’ together with 10 μL of Passive Lysis Buffer 2X (PLB 2X; Promega, Milan, Italy) to permeabilize yeast cells. The firefly luciferase activity was then detected measuring luminescence after adding 10 μL of luciferase substrate (Bright-Glo Luciferase Assay System; Promega). Optical density (OD) was measured at 600nm and used as normalization factor to obtain Relative Light Units, followed by blank subtraction. The analysis was performed using the microplate reader Infinite M200 (Tecan, Milan, Italy). Blank controls were also included.

### Phylogenetic analysis

p53 protein sequences were downloaded from OMA browser [38] based on the latest dataset. The multiple sequence alignment was performed using the alignment editor BioEdit v7.1.11 and visualized in Jalview [39]. The percentage of identity matrix was calculated using the software Clustal Omega [40]. The freeware MEGA6.0 (molecular evolutionary genetics analysis) tool [41] was used to infer radial phylogenetic trees based on the multiple sequence alignments. Phylogenetic analysis was performed using the neighbor-joining (NJ) method, and confidence limits of branch points were estimated by 1,000 bootstrap and Poisson substitution model. A 50% cut-off value for consensus tree was used to collapse nodes and re-rooting the tree. A species phylogenetic tree was originated using the NCBI Taxonomy Browser and visualized using MEGA6.0. The tanglegram algorithm, implemented in Dendroscope 3 [42] was used to compare the phylogenetic trees.

## Results

### p53 sequence evolution

**Fig 1:**
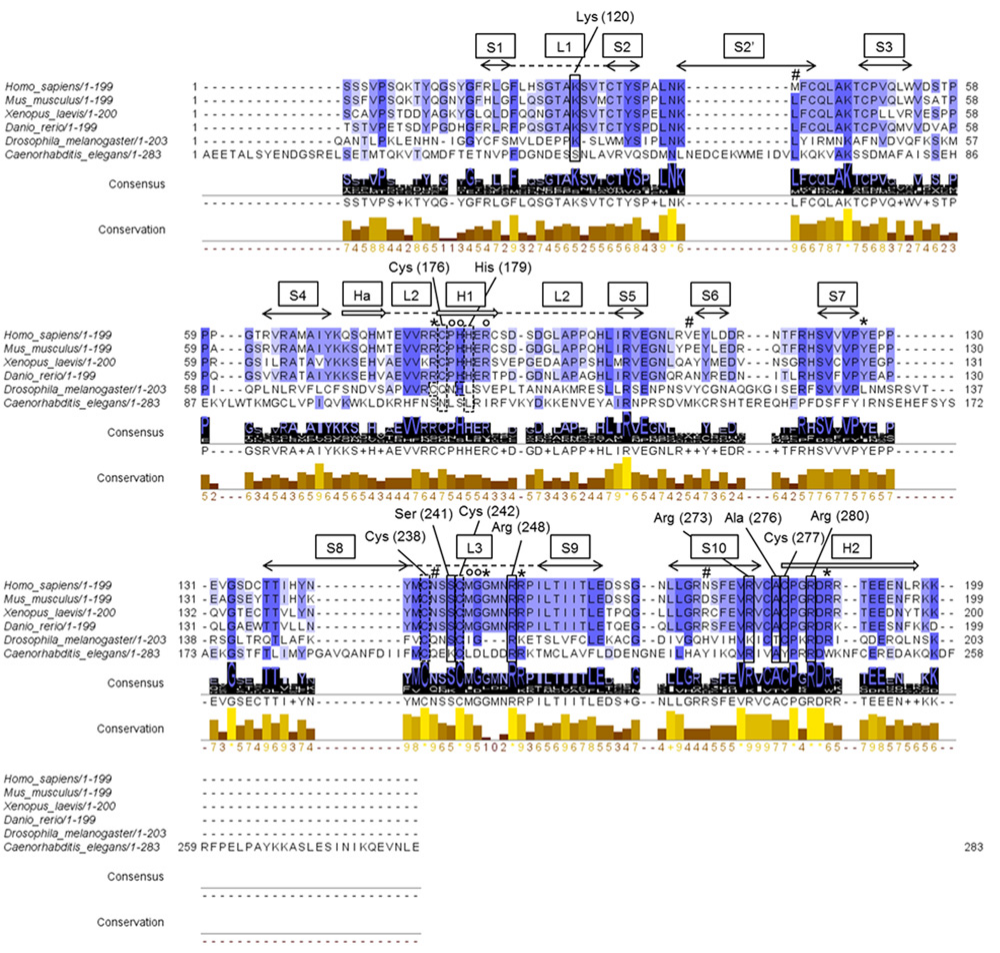
p53 DNA binding domain sequence alignment of Homo sapiens, Mus musculus, Xenopus laevis, Danio rerio, Drosophila melanogaster and Caenorhabditis elegans. Sequences were aligned using ClustalW tool [74] and the alignment was visualized using Jalview (http://www.jalview.org) [39]. The three shades of blue highlight different percentage agreement, respectively from darker to lighter in this order: >80%, >60%, >40%. A percentage agreement ≤40% is not highlighted. The alignment shows the conservation of human functional residues involved in DNA-protein interaction (solid box), zinc-binding (dashed box), DBD stability (asterisk, *), protein-protein interface (empty circle, #) and DBD thermostability (hash mark, #). Human zinc-binding and DNA-binding residue positions are also typed. On the top of the alignment, human p53 Loop, Sheet, Helix motifs (L, dashed lines; S, left-right double arrows; H, two-dimensional arrows) are also presented. Conservation and consensus graphs are shown. Conservation is visualized as a histogram with the relative score for each column. Conserved columns are indicated with an asterisk, and columns with mutations, where all properties are conserved, are marked with a plus. Consensus is displayed as the percentage of the modal residue per column. The plus symbol is used instead of displaying multiple characters in a single character space. A consensus logo is also generated and the scale of the letter is in agreement with the conservation of the residues.

We generated an updated comparative analysis of 47 p53 protein sequences in relation to metazoan phylogeny using the online OMA browser [38] (S1 File). Sequence alignment, pairwise comparison of sequence identity and the conservation of critical amino acids for DNA contact, oligomerization or thermodynamic stability [43] [44] were also performed on p53 sequences from *Homo sapiens* (Hs_p53, human p53) and commonly used animal models *Mus musculus* (Mm_p53, mouse p53), *Xenopus laevis* (Xl_p53, frog p53), *Danio rerio* (Dr_p53, zebrafish p53), *Drosophila melanogaster* (Dm_p53, fruit fly p53) and *Caenorhabditis elegans* (Cep-1, round worm p53) (Fig. 1 and S1 File) (Table 1 and 2). As expected, Hs_ and Mm_p53 share the highest overall identity (~78%), while for Dr_ and Xl_p53 the identity decreases to ~50% for all pairwise comparisons, whereas Dm_p53 and Cep-1 are the most divergent p53 proteins relative to chordates examined and share also less than 20% identity (Table 1A). The percentage of identity was higher considering exclusively the DBD (Table 1B and Fig. 1). The C-terminal region exhibited higher level of conservation respect to the N-terminal portion (Table 1C, Table 1D, and S1 File).

**Table 1:** Percentage of identity matrix of p53 proteins.

A) whole sequence	Hs_p53	Mm_p53	*Xl*_p53	Dr_p53	Dm_p53	Cep-1
Hs_p53	100.00	77.78	52.94	51.40	19.82	16.02
Mm_p53	77.78	100.00	53.39	52.84	21.26	17.83
Xl_p53	52.94	53.39	100.00	55.79	23.55	19.53
Dr_p53	51.40	52.84	55.79	100.00	21.74	18.08
Dm_p53	19.82	21.26	23.55	21.74	100.00	18.50
Cep-1	16.02	17.83	19.53	18.08	18.50	100.00
B) DBD sequence	Hs_p53	Mm_p53	Xl_p53	Dr_p53	Dm_p53	Cep-1
Hs_p53	100.00	88.44	67.34	69.85	24.23	17.09
Mm_p53	88.44	100.00	67.84	71.36	24.23	18.59
Xl_p53	67.34	67.84	100.00	70.85	24.62	13.50
Dr_p53	69.85	71.36	70.85	100.00	24.74	16.08
Dm_p53	24.23	24.23	24.62	24.74	100.00	13.30
Cep-1	17.09	18.59	13.50	16.08	13.30	100.00
C) N-terminal sequence	Hs_p53	Mm_p53	Xl_p53	Dr_p53	Dm_p53	Cep-1
Hs_p53	100.00	53.49	29.85	18.33	1.56	16.30
Mm_p53	53.49	100.00	27.27	24.14	3.28	20.93
Xl_p53	29.85	27.27	100.00	11.76	15.69	10.45
Dr_p53	18.33	24.14	11.76	100.00	11.11	15.00
Dm_p53	1.56	3.28	15.69	11.11	100.00	13.70
Cep-1	16.30	20.93	10.45	15.00	13.70	100.00
D) C-terminal sequence	Hs_p53	Mm_p53	Xl_p53	Dr_p53	Dm_p53	Cep-1
Hs_p53	100.00	67.90	42.50	38.10	9.52	8.89
Mm_p53	67.90	100.00	38.16	27.50	5.00	4.94
Xl_p53	42.50	38.16	100.00	45.56	11.63	3.33
Dr_p53	38.10	27.50	45.56	100.00	8.79	9.09
Dm_p53	9.52	5.00	11.63	8.79	100.00	11.58
Cep-1	8.89	4.94	3.33	9.09	11.58	100.00

The identity matrix values were obtained using the Clustal Omega tool (https://www.ebi.ac.uk/Tools/msa/clustalo/) based on the full length sequence (A); the DBD (B); the N-terminal region (C) or the C-terminal region (D) of *Homo sapiens* (Hs_p53), *Mus musculus* (Mm_p53), *Xenopus laevis* (Xl_p53), *Danio rerio* (Dr_p53), *Drosophila melanogaster* (Dm_p53) and *Caenorhabditis elegans* (Cep-1) p53 proteins.

**Table 2:** Conservation of key p53 functional residues.

A)	DNA-protein interaction						
Hs_p53	Lys120	Ser241	Arg248	Arg273	Ala276	Cys277	Arg280
Mm_p53	-	-	-	-	-	-	-
Xl_p53	-	-	-	-	-	-	-
Dr_p53	-	-	-	-	-	-	-
Dm_p53	-	-	-	Lys	Thr	-	-
Cep-1	Ser	Lys	-	-	-	Tyr	-

B)	Zn^2+^ binding sites			
Hs_p53	Cys176	His179	Cys238	Cys242
Mm_p53	-	-	-	-
Xl_p53	-	-	-	-
Dr_p53	-	-	-	-
Dm_p53	-	-	-	-
Cep-1	Asn	Leu	-	-

C)	protein-protein interaction				
Hs_p53	Pro177	His178	Arg181	Met243	Gly244
Mm_p53	-	-	-	-	-
Xl_p53	-	-	-	-	-
Dr_p53	-	-	-	-	-
Dm_p53	Gln/Asn	Asn/-	Ser/Val	Ile	-
Cep-1	Leu	Ser	Ile	Leu	Asp

D)	DBD stability				
Hs_p53	Arg175	Tyr220	Gly245	Arg249	Arg282
Mm_p53	-	-	-	-	-
Xl_p53	-	-	-	-	-
Dr_p53	-	-	-	-	-
Dm_p53	- / N/A	Leu	N/A	Lys	-
Cep-1	Ser	Ile	Leu	-	Trp

E)	DBD thermostability			
Hs_p53	Met133	Val203	Asn239	Asn268
Mm_p53	Leu	Pro	-	Asp
Xl_p53	Leu	Ala	-	Arg
Dr_p53	Leu	Ala	-	Arg
Dm_p53	Leu	-	Gln	His
Cep-1	Leu	Met	Gln	Tyr

Presented is a summary for *Homo sapiens* (Hs_p53), *Mus musculus* (Mm_p53), *Xenopus laevis* (Xl_p53), *Danio rerio* (Dr_p53), *Drosophila melanogaster* (Dm_p53) and *Caenorhabditis elegans* (Cep-1). See also Fig. 1. Reported are residues involved in DNA-protein interaction (A); zinc-binding (B); protein-protein interactions (C); DBD stability (D) and DBD thermostability (E).

Many of the known p53 mutations hit the core domain and this further emphasizes the importance of the p53 sequence-specific transactivation function and hence of the DBD [45]. All residues that can establish direct contacts with RE sequences were invariant in the four chordate p53 proteins (Table 2A). Arg280, Lys120, Ala276 and Cys277 allow p53 binding to DNA bases within the major groove; whereas Ser241, Arg273, Arg248, Lys120 and Ala276 form direct contacts to the DNA backbone [44]. In Dm_p53 most of the residues fundamental for the DNA contact are also conserved (Table 2A), with the exception of Arg273, that is replaced by the corresponding Lys259, and of Ala276, replaced by Thr262, as previously reported [36] [46] [45]. Arg248, Arg273, Ala276 and Arg280 appeared to be conserved even in Cep-1 (Table 2A), highlighting the functional importance of these residues for DNA binding specificity, thus supporting previous data [46] [45] [37]. Looking at the conservation of zinc coordinating residues [35] [37], residues important for DBD stability [36] [44] and residues involved in the non-polar protein-protein interface [45] [44], we noticed that, among the chordates studied, all these residues are conserved (Fig. 1 and Table 2). Many of them are also conserved in Dm_p53 whereas Cep-1 was the most divergent sequence. [35] [37] [44] [45] [36]. The residues Arg175, Met243, Gly245, Arg248, Arg249, Arg273, and Arg282 are classified as either contact residues or residues that influence thermo-stability. Moreover, Met133, Val203, Asn239 and Asn268 are important for thermo-stability of the human p53 core domain, as specific mutations at these residues can produce a ‘‘super-stable” p53 form [44] [47]. Surprisingly, in our alignment analysis these residues are not highly conserved among chordates, with the only exception of Asn239. Curiously, a methionine at position 133, corresponding to the start codon for ΔN133 p53 isoform, is present only in the human sequence, substituting a leucine that is present in all the other five species analyzed (Fig. 1 and Table 2E). This indicates a recent acquisition of ΔN133 isoform expression in the functional evolution of the p53 transcriptional network [48]. Lastly, human Val203 and Asn268 are replaced by alanine and arginine, respectively, in both Dr_ and Xl_p53.

### Yeast as a tool to investigate p53 functional evolution

Yeast-based transactivation assay was carried out using the six p53 proteins in Fig. 1 and a total of ten luciferase reporter strains, of which five are based on natural human p53 REs and five are variations of the p53 consensus sequences (Table 3) [14] [16]. This panel of sequences was chosen taking into account the p53 consensus RE matrix shared by p53 proteins from different species [45] to scan a wide range of DNA binding affinity and transactivation potential for human p53, and to include both canonical and non-canonical REs [16]. All REs are placed in the same chromatin landscape, hence a matrix of results could be obtained where differences in transactivation potentials are directly dependent on the nature of the RE sequence, the type of p53 protein being expressed, the level of expression of the p53 proteins, and the growth temperature of yeast strains [27].

**Table 3:** List of the consensus and natural RE sequences used in our experiments.

CONSENSUS SEQUENCE	RRRCWWGYYY-RRRCWWGYYY	Kd
CON1	GGGCATGTCC-GGGCATGTCC	-7.56
CON2	GGGCTAGTCC-GGGCTAGTCC	-6.82
CON3	GGGCAAGTCC-GGGCAAGTCC	-7.19
CON-J	GGGCTAGTCC-GGGCAC - - - -	-6.70
CON-K	GGGCATGTCC- tGttTTGTCC	-6.48

NATURAL HUMAN p53 RE	RRRCWWGYYY-RRRCWWGYYY	Kd
p21	GAACATGTCC-cAACATGTTg	-7.11
PUMA	ctGCAAGTCC-tGACTTGTCC	-6.86
MDM2 P2 (RE1 + RE2)	GGtCAAGTTC-AGACAcGTTCn_17_GAGTAagTCC-tGACTTGTCT	N/A
MMP2	AGACAAGCCT-GAACTTGTCT	-7.01
BAI1	tGGC—TGCCT-GGACATGTTC	-6.31

Reported are i) the RE name, ii) the nucleotide sequence with relative position of p53 monomer-binding sites (arrows) and iii) the calculated dissociation constant based on [49]. Non-consensus bases are reported in lowercase. n = spacer length.

cDNAs for the six p53 proteins were cloned into yeast expression vectors using gap repair approaches (see Materials and Methods). To perform the functional assay, transformants were incubated for six hours in media containing two different concentrations of galactose, to achieve moderate (0.008%) and high (0.064%) p53 cDNA expression (see Materials and Methods).

### A 10 RE panel to investigate the impact of different features on p53 transactivation potential

**Fig 2:**
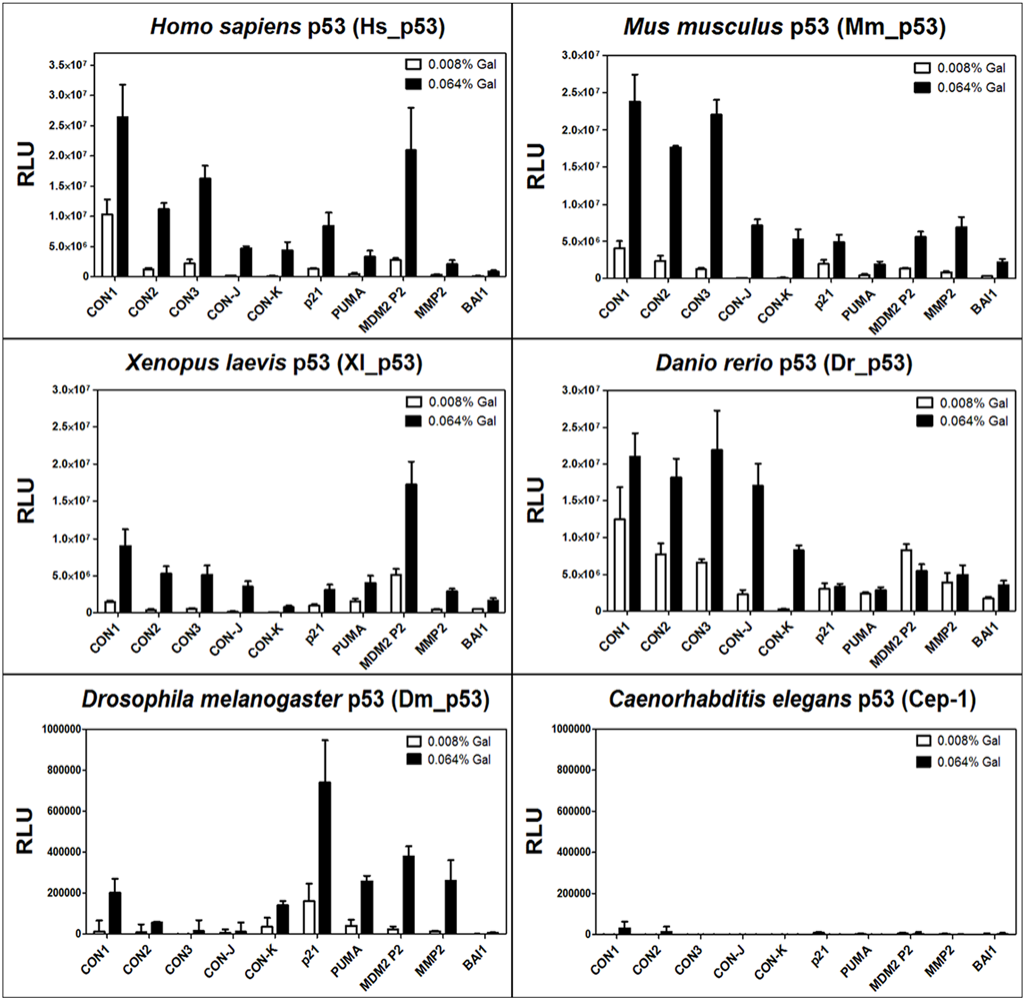
Yeast-based transactivation analysis of p53 proteins towards a 10 RE panel. Results were obtained as indicated in Material and Methods. Presented are the averages of relative light units (RLU), defined as the light unit normalized by the optical density at 600nm and after the subtraction of the empty values, *i*.*e*. the p53-independent expression of the reporter. Error bars plot the standard deviation of at least four independent replicates. The activity of p53 proteins from the indicated species was measured after 6 hrs incubation at 30°C in media containing two concentrations of galactose (0.008% and 0.064%), that regulates the expression of the p53 transgene. Results obtained with fruit fly p53 and worm p53 are plotted with a different scale due to the low induction of the reporter. The p53 REs used are indicated in the X-axis (see Table 3).

**Fig 3:**
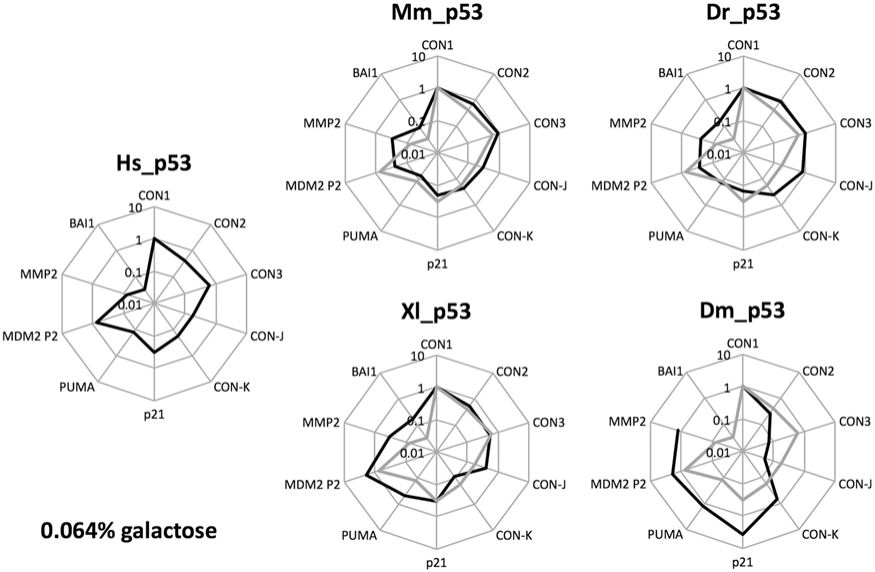
Evolutionary changes in p53 transactivation specificity. Transactivation potentials for the ten REs tested are presented as radar plot graphs in Log_10_ scale, relative to the results obtained with CON1 (set to 1). The resulting images represent the transactivation specificity for the indicated p53 proteins and that of human p53 is also overlaid (gray line) in every panel to facilitate comparisons. The yeast-based transactivation results at 0.064% of galactose concentration were used. Results obtained at 0.008% galactose are presented in S1 File.

The relative transactivation potential was measured for each protein towards the 10 REs (Fig. 2) and changes in relative transactivation specificity were compared (Fig. 3). This latter comparison is largely independent from relative p53 protein expression, stability or nuclear localization and is the focus of this study. Consensus (CON) REs are artificial REs chosen to investigate more directly the impact of sequence changes in the CWWG core motif (TA, AT or AA) (Table 3 with relative predicted K_d_). The non-canonical REs CON-J and CON-K were tested to examine protein capacity towards low affinity REs [49]. In particular, CON-J is composed of a 1/2 site contiguous to a 1/4 site (defined as 3/4 site), whereas CON-K consists of a 1/2 and 1/4 site separated by a 5-nt spacer (Table 3). The functional ranking of these REs with Hs_p53 (Fig. 2) confirmed previous results [16] [30]. In particular, CON1 was the most responsive followed by CON3 and CON2, while CON-J and CON-K were the weakest particularly at the lower galactose concentration. As expected, the highly conserved Mm_p53 showed comparable results with respect to the human ortholog. Xl_p53, and particularly Dr_p53, instead exhibited reduced discrimination towards the REs, more evident at the higher expression level (Fig. 2). CON-K remained the weakest RE. Dm_p53 showed very low level of activity compared to the chordate p53s tested. Surprisingly, responsiveness of CON-K was nearly comparable to CON1. Cep-1, the most evolutionary divergent p53 protein examined, was instead not functional towards all consensus REs tested.

Analysis was further extended to five p53 REs derived from natural human p53 target genes (p21, PUMA, MDM2 P2, MMP2, and BAI1; Table 3). These REs contain mismatches from the consensus (except for MMP2) and differ among each other also at the Rs and Ys flanking the CWWG core. BAI-1 is a 3/4 site whereas MDM2 comprises two REs (P2) separated by a spacer that can act cooperatively in transactivation [16]. The predicted K_d_s are in the same range as for the consensus REs (Table 3). MDM2 P2 was the most responsive natural RE to Hs_p53 (Fig. 2), followed respectively by p21, PUMA and MMP2, and BAI1 as the weakest. Surprisingly, Mm_p53 was particularly active towards MMP2 but less towards MDM2 P2 (Fig. 2). Xl_p53 showed a transactivation pattern comparable to that of Hs_p53 with the exception of p21 RE, which was less active at the higher level of expression. Dr_p53 confirmed a lower degree of discrimination among REs and high activity at lower p53 expression. With the exception of BAI1, transactivation was not correlated with galactose concentrations. Dm_p53 exhibited again a low overall transactivation potential, was completely inactive towards BAI1, while p21 was the most responsive RE. Cep-1 was also inactive towards all the natural human p53 REs tested (Fig. 2).

### Widespread evolutionary divergence of p53 transactivation specificity

To compare directly relative changes in transactivation specificity for each p53 protein examined, we summarized the results from the functional assay using radar charts (Fig. 3). As a matter of fact, relative light unit (RLU) values cannot be directly compared given the lack of quantification of nuclear p53 proteins level. A visualization of relative transactivation specificity was obtained setting as 1 (100%) the results obtained for the most responsive RE with human p53 (CON1) at either level of expression. The result for human p53 was then overlaid with those obtained for the other p53 proteins (Fig. 3).

The profile of transactivation specificity was consistent for the data obtained with two concentrations of galactose inducer (Fig. 3 and S1 File), although weaker REs, such as CON2, CON-J or BAI-1 were more responsive at higher galactose concentration, possibly reflecting differences in p53 oligomeric state that can be affected by protein concentration. While the general conservation of transactivation potential is apparent, as most of the REs were active with the different p53 proteins, the radar plots indicated that each p53 protein tested exhibited a unique profile of transactivation specificity. Mm_p53 and Xl_p53 were the most similar to Hs_p53 (S1 File). However, Mm_p53 and Xl_p53 exhibited an apparent change in relative transactivation specificity that resulted in lack of discrimination between PUMA and BAI1 RE and higher relative activity towards MMP2. Dr_p53 profile resembled more the Hs_p53 one at low galactose concentration (S1 File), but had no apparent discrimination between the REs at higher galactose dose. Dm_p53 showed a unique pattern of transactivation specificity with high activity for all natural REs except for BAI-1.

### Xenopus laevis p53 is highly temperature sensitive

**Fig 4:**
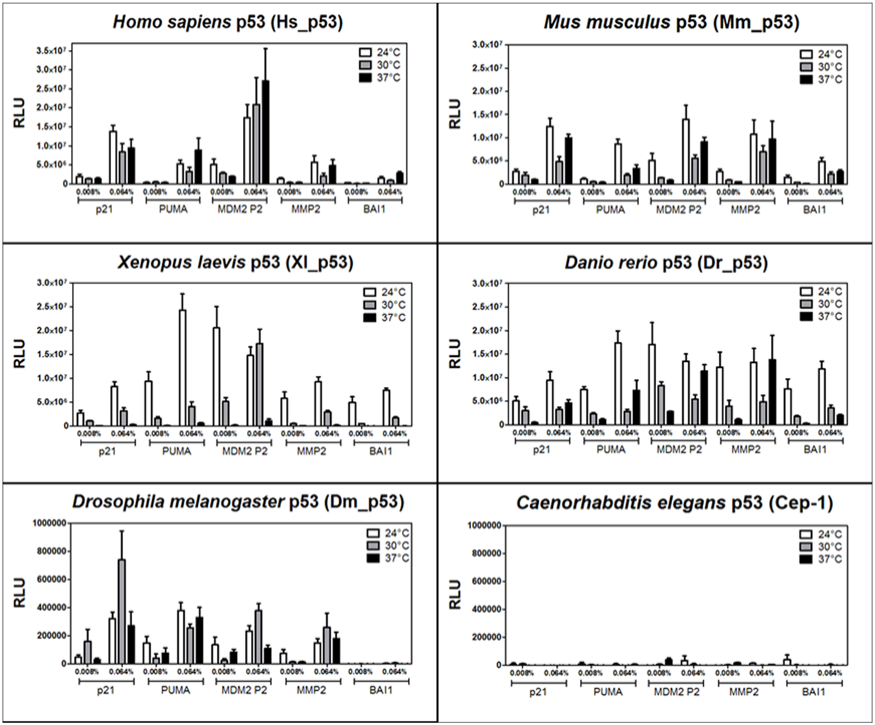
Influence of temperature on the transactivation potential of p53 proteins. The ability of p53 proteins to transactivate from the five natural REs at different expression levels was measured culturing yeast cells at 24°C (white bars) or 37°C (black bars) in media containing the indicated amount of galactose. Data obtained at 30°C (Fig. 2) were re-plotted (gray bars) for comparison. The experiments were performed and data were analyzed and presented as for Fig. 2.

Since we studied p53 proteins derived from either homeothermic (mouse and human) or poikilothermic (frog, zebrafish and fly) animals, we decided to carry out the yeast transactivation assay both at 37°C and 24°C using the five natural p53 REs (see Materials and Methods). Results were then compared to the previous data acquired at 30°C (Fig. 4). Hs_p53 had a comparable activity at all three different temperatures tested. Unexpectedly, Mm_p53, showed some temperature sensitivity, especially at low expression levels. Dr_p53 was also temperature sensitive particularly at lower protein expression (Fig. 4), where a ‘‘ladder” effect consisting in a progressive decrease in transactivation capacity, was observed with increasing temperatures. No significant, temperature-induced differences were recorded with Dm_p53. Cep-1 remained inactive also at 24°C and 37°C.

Xl_p53 was the most sensitive to temperature for all the REs tested and with both galactose concentrations used, consistent with previous reports [50], whereas a significant gain in transactivation capacity was observed at 24°C (Fig. 4).

### A chimeric transactivation domain strongly enhances Drosophila melanogaster p53 transcriptional activity in yeast

**Fig 5:**
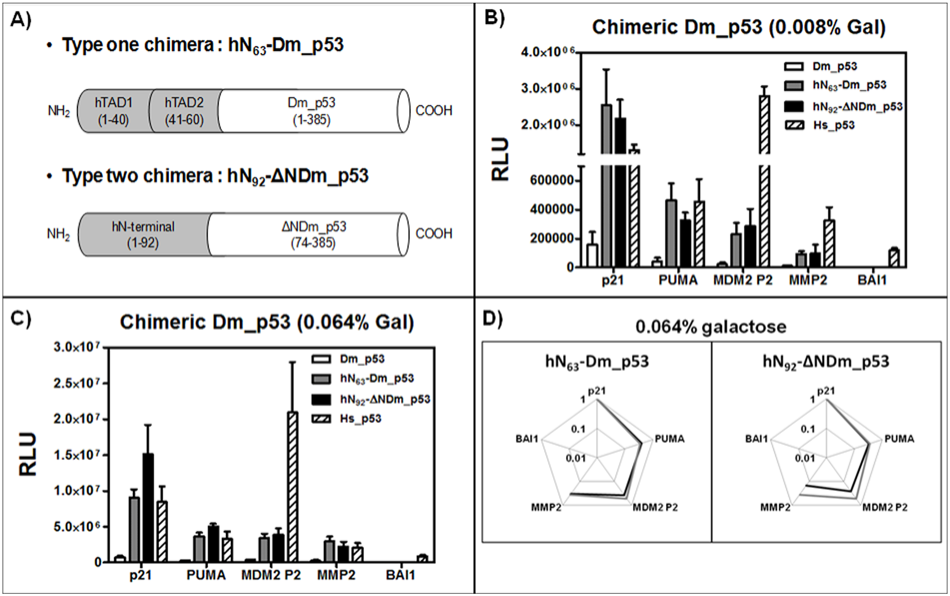
Chimeric Dm_p53 exhibited higher transactivation potential but no changes in transactivation specificity. **A)** schematic view of the two types of chimeric constructs tested, as described in Material and Methods. **B, C)** Transactivation activity of Dm_p53, hN_63-_Dm_p53 and hN_92-_ΔNDm_p53, and Hs_p53 proteins on five human natural p53 binding sites (see Table 3). Two galactose concentrations (0.008% and 0.064%) were tested. **D)** Radar plot charts in Log_10_ scale of relative transactivation potential of chimeric Dm_p53 (black line) relative to the results with the p21 RE (set to 1). Results with non-chimeric Dm_p53 are overlaid (gray line).

As mentioned above, despite the low level of conservation in the DBD, biochemical assays suggest that both Cep-1 and Dm_p53 can bind to canonical p53 REs *in vitro* [36] [45]. Since the N-terminal transactivation domain is poorly conserved, and given that different types of transactivation domains may function less effectively in yeast cells [51], we generated chimeric constructs to provide an acidic transactivation domain to these p53 proteins derived from protostome animals. Hence, we created chimeric proteins for both Dm_p53 and Cep-1, as described in Materials and Methods. In these constructs (see also Fig. 5A), human p53 N-terminal portions were either used to replace the N-terminal of Cep-1 and Dm_p53 or added at the 5’ of full-length p53 from both species. These chimeras were tested towards the five natural human p53 REs.

The two Dm_p53 chimeric proteins showed much higher transactivation capacities compared with the non-chimeric counterpart, reaching similar transactivation values as obtained with the p53 proteins from chordates (Fig. 5B and 5C). As a comparison, human p53 activity was also included. Dm_p53 chimeric proteins possess a transactivation potential similar to human p53 towards the REs p21, PUMA and MMP2. Interestingly, Hs_p53 is instead more active than the chimeric Dm_p53s towards MDM2 P2 and BAI1, indicating that the responsiveness of these chimeric constructs is likely more dependent on the affinity of their DBDs towards the REs. The human transactivation domain increased Dm_p53 transactivation capacity in our system without impacting the overall transactivation potential. Indeed, both type of chimeric constructs showed similar transactivation specificity, comparable also to the non-chimeric Dm_p53, as highlighted by the radar plots (Fig. 5D). Taken together, these findings indicate that chimeric strategy can be used in our system to study TFs specificity.

Four different chimeric Cep-1 constructs were tested (see Materials and Methods) but all remained inactive in the transactivation assay (data not shown), as the non-chimeric construct.

### Cep-1 is active towards the ced-13 p53 RE in yeast

**Fig 6:**
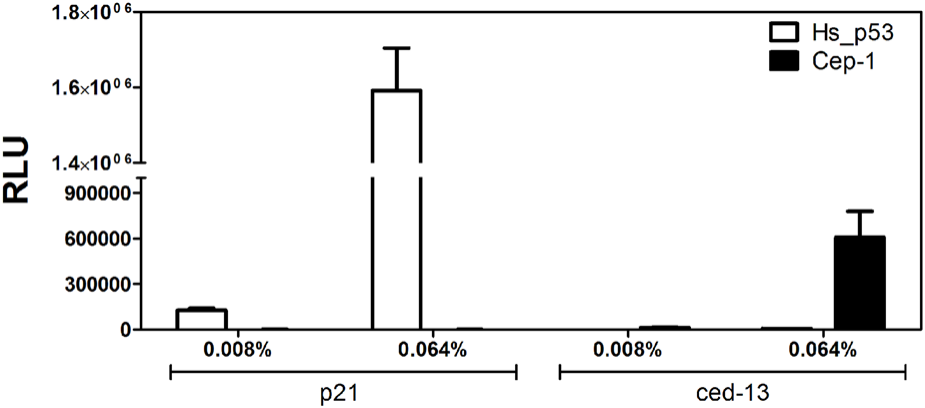
Wild type Cep-1 is functional on a *C*. *elegans* natural RE. Transactivation activity of Hs_p53 and Cep-1 proteins on the human natural p21, used as a positive control (see Table 3), and the worm natural ced-13 binding sites. Results were obtained as indicated for Fig. 2, except for a 4-hour incubation time in galactose-containing media. Two galactose concentrations (0.008% and 0.064%) were tested. *Caenorhabditis elegans* Cep-1 binding site of ced-13 is the following: AAACATGTTT(N)_28_AAACATGTTT.

We reasoned that one possibility for lack of Cep-1 activity could be ascribed to a markedly diverged DNA binding specificity of this protein. To date, no functional Cep-1 binding sites have been reported or annotated. Thus, we constructed a reporter strain (see Materials and Methods) based on the evidence of a potential p53-binding sites described in the promoter of ced-13, composed of two AAACATGTTT palindromic half sites separated by a 28nt spacer [28]. None of the REs previously tested in our work had these A/T-rich motif flanking the CWWG core. Surprisingly, while the strain was not responsive to Hs_p53 (as expected because of the long spacer) non-chimeric full-length Cep-1 led to modest but significant transactivation of the reporter (Fig. 6). To our knowledge, this is the first evidence of a functional binding site responsive to Cep-1 in transactivation assays. Hence, the lack of transactivation towards human or artificial REs by natural or chimeric Cep-1 proteins is likely to be dependent on a lack of affinity of the DBD. This result is in agreement with the hypothesis of co-evolution of p53 DBD and binding sites and warrants further investigation in the future.

## Discussion

A role of *cis*-regulatory elements (CREs) evolution and of co-evolution of CREs and TFs in determining phenotypic diversity among species have been recently emphasized [2]. Conserved enhancer elements that can be bound by TFs are thought to be functionally essential and are usually enriched in proximity of key target genes, such as those controlling body plan development. On the other hand, lack of conservation of TF binding sites do not necessary imply lack of function but rather can underlie diversity in the regulation of gene expression. For example, the loss of a DNA binding element can be counterbalanced by the gain of a different element acting on the same transcriptional start site(s) [52]. Importantly, TF binding sites are usually degenerated. Hence, the presence of TF binding sites within orthologous promoters can be conserved but the actual sequence of the elements can differ, resulting in variation in relative binding affinities that can play an important role in expression divergence between species [2] [16]. On the other end, evolutionary divergence or conservation of gene regulatory modules can be strongly influenced by the promoter context, *i*.*e*. the gene regulatory network impacting on the activation of a target promoter, or the integration of signals influencing biological outcomes [53]. Mobile repetitive elements can contribute to the spread of DNA binding elements, thereby contributing to the expansion of gene regulatory networks [24] [54]. Moreover, convergent evolution is another way to gain binding events in proximity of genes involved in the same functions or expressed in the same tissues between two organisms. A fascinating case linked p53-dependent regulation of genes involved in neuronal development with upstream insertion of specific transposons carrying p53 binding elements. The insertion of these elements convergently happened in both human and zebrafish genomes [55].

### Apparent evolutionary divergence of p53 transactivation specificity

We sought out a versatile experimental system that could evaluate the transactivation potential and specificity of p53 proteins and, in principle, of any sequence-specific transcription factor. We chose a well-established yeast-based assay that minimizes the impact of variables such as chromatin state, promoter landscape and cofactors’ influences. In fact, all REs are placed in the same genomic locus, at single copy, and at a target chromosomal site upstream of the same minimal core promoter in completely isogenic reporter strains. The tool is versatile also because the p53 protein of interest can be expressed at different levels through an inducible promoter. In addition the assay can be performed in a small volume format, potentially amenable to automation [27].

Given that presently our approach does not take into account relative nuclear protein levels for the different p53 proteins expressed in yeast, comparisons can be drawn on changes in transactivation specificity of a given p53 protein towards different REs (Fig. 3). The REs were chosen to sample a wide range of sequence variability (Table 3), DNA binding affinity [49] and transactivation potential with human p53 [16]. Some of them are considered non-canonical, as they do not provide contact sites for all four monomers of the p53 tetramer [13] [56].

In our analysis, mouse and human p53 show high level of conservation, that does not necessary preclude functionally relevant changes in protein structure and function. However, the mouse p53 DBD could be substituted by the human DBD in a chimeric transgenic model, without apparent phenotypic consequences [57]. Divergence is instead apparent for p53 REs between human and mouse genomes. Approximately two-thirds of the known human p53 binding sites are not alignable with their mouse counterparts, leading to a large divergence between the *cis*-regulatory networks, although the murine homologous genes still maintain the same function as in humans [52] [23]. Moreover, a class of p53 REs was identified in Alu elements and is thought to be primate-specific, an additional source of CRE differences between humans and mice [54]. Overall, all these findings support the idea of a different qualitative (target gene networks) and quantitative (strength or responsiveness) regulation of the p53 pathway between humans and rodents. Nevertheless, most p53-induced transcriptional responses, such as cell cycle arrest and induction of apoptosis, predate the common ancestor of human and rodents.

While there was undoubtedly a conservation of sequence-specific transactivation as most of the REs were responsive to all p53 proteins tested, with the exception of Cep-1, differences in transactivation specificities were apparent for each protein even in the comparison between Hs_ and Mm_p53 (Fig. 3). Xl_p53 was similar in terms of relative transactivation specificity to human and mouse p53 (Fig. 3 and S1 File). A consistent trend was observed for Xl_p53, Mm_p53 and particularly Dr_p53, which have higher relative transactivation potential towards lower affinity [49] (Table 3), non-canonical [16], or structurally more rigid [58] binding elements. In fact Dr_p53, especially when expressed at higher levels, lacked RE discrimination in terms of relative transactivation potential, a result not strictly related to saturating p53 protein level, as shown at 24°C. This peculiar transactivation capacity can be explained by the fact that although Dr_p53 is structurally and functionally similar to its human counterpart, it still retains some differences at key residues in the C-terminal helix, at the level of the tetramerization domain [59] [60].

Among the p53s tested in our assay, Dm_p53 was active only on a subgroup of REs.

### *Xenopus laevis* and, to a less extent, *Danio rerio* p53 are temperature sensitive proteins

Thermodynamic stability has been extensively investigated for p53, in relation to its DNA-binding properties [20] [61] [47]. As a matter of fact, the melting temperatures (and consequently the stability) of p53 orthologs reflect the respective body temperature [61].

The transactivation assay confirmed severe temperature sensitivity for Xl_p53. Transactivation was nearly abolished when the growth temperature of yeast reporter strains was raised to 37°C. To note, these results are in agreement with previous observations that Xl_p53 is fully active at 30°C but inactive at 37°C [50]. Conversely, we also showed a clear gain in transactivation potential at 24°C. Indeed, *Xenopus laevis* standard laboratory rearing temperature is usually ~22–24°C [62] [63] and an increasing of temperature to 30°C is sufficient to activate a heat shock response [64] [65]. The observed Xl_p53 temperature sensitivity could suggest a possible mechanism evolved to inactivate p53 activity when environmental temperature increases.

Dr_p53 also showed some degree of temperature sensitivity that was appreciable especially at lower protein expression and with a concomitant gain at 24°C (Fig. 4).

Thus, we propose that our assay could be specifically employed for thermo-sensitivity studies and to investigate or identify mutations altering temperature-dependent transactivation of TFs.

### Chimeric Dm_p53 show higher transactivation potential without changes in specificity

*In vitro* EMSA assays showed that monomeric p53 proteins, even from distant species with overall low homology such as Cep-1 and Dm_p53, exhibit conserved DNA binding affinity (with similar dissociation constant) with respect to human p53 [61] [36] [66]. Crystal structure analysis overall confirmed this observation [67] [37]. However, the correlation between *in vitro* DNA binding affinity and transactivation potential for p53 family proteins can vary and recent data indicate that changes in the oligomeric state or in the cooperative interaction between DBD domains within a tetramer, induced by post-translational changes or due to sequence divergence, can strongly impact on transactivation specificity [14] [68] [69] [67].

Our results were not in agreement with these observations. Indeed, Dm_p53 exhibited weak transactivation activity towards some of REs tested, and Cep-1 was inactive with all the ten REs investigated (Fig. 2) It is worth noting that the assay we used does not directly measure DNA binding but rather p53-dependent modulation of transcription. This means that Dm_p53 and Cep-1 could in fact bind the REs but are inefficient or incapable to recruit transcriptional components essential for the activation of the minimal promoter driving the expression of the luciferase reporter. Earlier studies demonstrated that some classes of transactivation domains are not active in yeast [51] and this was also confirmed for human cardiac TFs [70]. To explore this possibility, and potentially solve this shortcoming of the experimental approach, we constructed chimeric proteins harboring the human p53 transactivation domains (hTADs) that are active in yeast and that either replaced the predicted corresponding N-terminal region or were added upstream the full-length sequence of Dm_p53 or Cep-1.

The activity of Dm_p53, in both chimeric proteins, was strongly enhanced. hTADs were sufficient to gain a transactivation capacity comparable to the human full length protein, even when just fused upstream to the Dm_p53 N-terminal domain. Importantly, the chimeric construct did not induce significant changes in transactivation specificity. Instead Cep-1 chimeric constructs remained inactive for transactivation. However we found wild type Cep-1 to be active towards its putative RE from the ced-13 gene (Fig. 6). These results pointed out that the sequence and structural differences between Cep-1 and the other chordate p53s are sufficient to impact its transcriptional activity, opening up possible future investigations about the evolutionary conservation of p53 DNA binding affinity and its coevolution with p53 binding sites and regulatory network. Of note, Cep-1 presents also marked structural and size differences in its C-terminal domain compared to the chordate p53 homologs. An oligomerization domain (OD) and a SAM domain are present at the C-terminal portion of Cep-1 protein, conferring a higher degree of protein stability than human p53 [61] [71].

In conclusion, the *cis*-regulatory network divergence could affect functional experiments when performed to examine the *in vivo* or *in vitro* effects of TF activation, deletion or a mutant TF. We propose that our yeast-based approach could be integrated in the plethora of tools commonly used in functional evolutionary studies as it allows robust assessment of relative transactivation specificity in a defined experimental set-up. Here we used, as a proof of concept, the tumor suppressor p53. Overall, we addressed differences in p53 transactivation capacity and specificity using p53 proteins derived from commonly used animal models. Unexpectedly, we observed widespread evolutionary divergence of p53 transactivation specificity, from Dr_p53 possessing similar transactivation potential towards different types of REs, to Hs_p53 exhibiting a wide range of transactivation potential, depending on the RE. These differences might be related to changes in the p53 transcriptional network and regulated functions in the course of metazoan evolution [36] [72] [73]. We could not investigate thoroughly the potential for p53 REs co-evolution in the genomes of the six-species analyzed, which could constitute a development of this approach. In this regard, as an example we report results obtained on two putative Xl_p53 REs (S1 File). We performed an *in silico* pattern search analysis that led us to identify potential p53 REs in the promoter region of mdm2 and cdkn1a genes of *Xenopus laevis* (S1 File). This mdm2 RE was similarly responsive to both Hs_p53 and Xl_p53, whereas cdkn1a RE was inactive. These explorative results established no clear evidence for p53 protein and binding site co-evolution among Hs_p53 and Xl_p53. In the future, we also plan to compare p53 proteins derived from a larger range of species, to investigate how p53 transactivation specificity has been shaped during evolution and after the emergence of the three-gene p53 family.

## Supporting Information

S1 File Supporting Information Figures, Tables, and Legends. Contains Supporting Information on p53 protein sequence evolution.
(ZIP)
