# Supplementary material for: Evolution of p53 Transactivation Specificity through the Lens of a Yeast-Based Functional Assay
Source: PLoS One. 2015 Feb 10;10(2):e0116177. doi: 10.1371/journal.pone.0116177 (PMC4323202; doi:10.1371/journal.pone.0116177)
Supplement: S1 File — Contains Supporting Information on p53 protein sequence evolution. (ZIP) [file pone.0116177.s001.zip › Supporting Information.docx]

**Supporting Information**

**p53 *sequence evolution: an update based on 47 species***

To generate an updated comparative p53 protein sequence analysis in relation to metazoan phylogeny we used the online OMA browser [1]. We obtained 47 annotated p53 protein sequences, the majority (42) belonging to the Chordata phylum, including species belonging to the subphyla Vertebrata (39), Tunicata (2) and Cephalochordata (1). Other four proteins were from the Arthropoda phylum (*Ixodes scapularis, Daphnia pulex, Drosophila melanogaster* and *Pediculus humanus*) and only one from the Nematoda phylum (Cep-1, *Caenorhabditis elegans*) (Figure S1). As expected, the region corresponding to the DNA binding domain (DBD, residues 94-292 for the human p53) shows the highest level of sequence conservation, consistent with its essential role for p53 function [2] [3] [4]. A percentage of identity matrix was also generated (Table S1) with a level of sequence identity ranging from over 95% to ~15%. In particular, sequences belonging to the superfamily Hominoidea show more than 95% identity with the human counterpart (Table S1) and, overall, all the mammalian p53 proteins exhibit more than 60% identity with human p53. Interestingly, Cep-1 exhibited less than 20% identity with any other p53 sequence examined. Phylogenetic trees were inferred using the free Molecular Evolutionary Genetics Analysis freeware (MEGA) [5], based on the alignment of both full-length and DBD p53 proteins sequences (Figure S2A&B). These phylogenetic reconstructions are consistent with previous published data [4] [2] [6] (Figure S2C). Indeed, gene phylogenies based on the full sequence or on the core domain of p53 produced highly similar tree topologies relative to organismal phylogeny based on taxonomic ranks, as shown in the tanglegram analysis (Figure S3). This confirms that p53 constitutes a suitable marker for high-rank phylogenetic reconstruction in metazoans.

**Supplementary Figure Legends**

Figure S1. Sequence alignment of 47 annotated p53 proteins. Sequences were obtained from the online OMA browser [1] and aligned using ClustalW tool [7]. The alignment was visualized using Jalview [8]. The three shades of blue highlight different percentage agreement, respectively from darker blue to lighter blue in this order: > 80%, >60%, >40%. $\boldsymbol{\leq}$40% percentage agreement is not highlighted.

Colored bars on top of the alignment depict the three main human p53 domains: the N-terminal domain (yellow; 1-92 human residues), the DNA binding domain (red; 94-292 human residues) and the C-terminal domain (blue; 304-393 human residues). The alignments in fasta format are also provided.

**Figure S2**. **Radial phylogenetic trees of the 47 p53 proteins.** Amino acid sequences were inferred by Neighbor-Joining clustering method and 1,000 bootstrap iterations, using the MEGA6.0 software [5]. Nodes with bootstrap consensus below 50% were collapsed. (A) Phylogenetic tree obtained from the alignment of full-length p53 sequences. (B) Phylogenetic tree obtained from the manually corrected ClustalW alignment of p53 DNA binding domains. (C) Species tree obtained from the NCBI taxonomy browser.

**Figure S3**. **Comparison of the phylogenetic trees**. The comparison was inferred from multiple sequence alignment of the full-length (A) and of the DBD (B) of the 47 p53 proteins with the species tree generated from the NCBI taxonomy browser. Corresponding species are linked by a line highlighting topological similarity between the two trees. The comparison was obtained using the tanglegram algorithm in Dendroscope3 [9].

Figure S4. p53 protein sequence alignment of *Homo sapiens*, *Mus musculus*, *Xenopus laevis*, *Danio rerio*, *Drosophila melanogaster* and *Caenorhabditis elegans*. The sequences were aligned using ClustalW tool [7] and the alignment was visualized using Jalview [8]. The three shades of blue highlight different percentage agreement, respectively from darker blue to lighter blue in this order: > 80%, >60%, >40%. $\boldsymbol{\leq}$40% percentage agreement is not highlighted.

On top of the alignment human p53 sub-domains are shown as: transactivation domain 1 (TAD1, 1-40), transactivation domain 2 (TAD2, 41-60), proline rich region (PR, 64-92) in the N-terminal domain (yellow); the DNA binding domain (red); nuclear localization sequence (NLS, 304-322), tetramerization/oligomerization domain (OD, 325-356), regulatory C-terminal (357-393) domain in the C-terminal domain (blue).

Conservation and consensus graphs are also depicted. Conservation is visualized as a histogram with the relative score for each column. Conserved columns are indicated by an asterisk, and columns with mutations where all properties are conserved are marked with a plus. Consensus is displayed as the percentage of the modal residue per column.

The plus symbol is used instead of displaying multiple characters in a single character space. A consensus logo is also generated and the scale of the letter is in agreement with the conservation of the residues.

**Figure S5**. **Radar plot graphs of relative p53 transactivation specificity.** Transactivation potential for the ten REs tested is presented in Log_10_ scale, relative to the results obtained with CON1 (set to 1). The yeast-based transactivation results at 0.008% galactose were used. As for Figure 3, the images represent the transactivation specificity for the indicated p53 proteins and that of human p53 is overlaid (gray line) in all plots to facilitate comparisons. Results with Dm_p53 were not plotted as the transactivation values were similar to those obtained with the empty vector for several REs (Figure 2).

**Figure S6. Quantification of the similarities in Radar Plot data.**

A) Cluster analysis of radar plot data presented in Figure 3 was performed using the R statistical software package ‘‘pvclust’’, in order to quantify the differences we observed in species-specific transcriptional activity. Hierarchical clustering assigns objects (heat map columns) to clusters with the intention to combine similar objects within a common cluster and dissimilar objects within different clusters using pairwise distances thus allowing to measure the distances between the objects (Height). To validate the results of clustering, a bootstrapping approach was used, which estimates the stability of resulting clusters by repeatedly applying the hierarchical clustering to subsets of the data for *n* times (*n*=100). The stability of such clustering can be described by **a**pproximately **u**nbiased (au) *p-value* (values at branches). (B) Graphic representation of correlation matrix used to generate the dendrogram in panel A; color represents the intensity of correlation coefficient; for each paired comparison the *p-value* is reported in the square.

**Figure S7. Hs_p53 and Xl_p53 transactivate similarly from Xenopus endogenous REs.**

Transactivation capacity of Hs_p53 and Xl_p53 was compared using two newly constructed yeast reporter strains containing putative natural Xl RE derived from the p21 and MDM2 genes (see Table S2 for sequence, genomic position, RE structure and comparison with RE from the ortholog human gene). Two different galactose concentrations were used, as indicated. Cells were culture in galactose media for 4 hours. Results are plotted as average light units normalized based on the optical density. Error bars plot the standard deviations of four biological replicates.

**Supplementary Tables**

**Table S1. Percentage identity matrix of the 47 p53 proteins.** Percentage values were obtained using the Clustal Omega tool [10] based on the full-length sequences. –Due to its size, Table S1 is presented as a separate excel file-

**Table S2. p53 REs in orthologs target genes.**

| **Gene name** | **Species** | **RE sequence^** | **Location from TSS** | **RE type*** |
| --- | --- | --- | --- | --- |
| p21 | Homo sapiens | GAA**CATG**TCCcAA**CATG**TTg | -2189 | FS |
|  | Xenopus laevis | AAG**CATG**TCCn_10_AAG**CTTG**TTTGAACT | -2592 | FS |
|  |  |  |  |  |
| MDM2 | Homo sapiens | GGt**CAAG**TTCAGA**CAcG**TTCn_17_  GAGtTAaGTCCtGA**CTTG**TCT | +747 | 2x FS |
|  | Xenopus laevis | GGt**CATG**CTCAGA**CATG**CaCn_14_  GGt**CTAG**CCCtGA**CTTG**TCT | +750 | 2x FS |

^ genome sequence from Ensembl.

* FS= full site;

The red font highlights the sequence divergence; lowercase indicates the mismatches from consensus; bold indicates the putative CWWG core motif; n = spacer length

**Supporting Information References**

1. Altenhoff AM, Schneider A, Gonnet GH, Dessimoz C (2011) OMA 2011: orthology inference among 1000 complete genomes. Nucleic Acids Res 39: D289-294.

2. Belyi VA, Ak P, Markert E, Wang H, Hu W, et al. (2010) The origins and evolution of the p53 family of genes. Cold Spring Harb Perspect Biol 2: a001198.

3. Dotsch V, Bernassola F, Coutandin D, Candi E, Melino G (2010) p63 and p73, the ancestors of p53. Cold Spring Harb Perspect Biol 2: a004887.

4. Lu WJ, Amatruda JF, Abrams JM (2009) p53 ancestry: gazing through an evolutionary lens. Nat Rev Cancer 9: 758-762.

5. Tamura K, Stecher G, Peterson D, Filipski A, Kumar S (2013) MEGA6: Molecular Evolutionary Genetics Analysis version 6.0. Mol Biol Evol 30: 2725-2729.

6. Rutkowski R, Hofmann K, Gartner A (2010) Phylogeny and function of the invertebrate p53 superfamily. Cold Spring Harb Perspect Biol 2: a001131.

7. Larkin MA, Blackshields G, Brown NP, Chenna R, McGettigan PA, et al. (2007) Clustal W and Clustal X version 2.0. Bioinformatics 23: 2947-2948.

8. Waterhouse AM, Procter JB, Martin DM, Clamp M, Barton GJ (2009) Jalview Version 2--a multiple sequence alignment editor and analysis workbench. Bioinformatics 25: 1189-1191.

9. Huson DH, Scornavacca C (2012) Dendroscope 3: an interactive tool for rooted phylogenetic trees and networks. Syst Biol 61: 1061-1067.

10. Sievers F, Wilm A, Dineen D, Gibson TJ, Karplus K, et al. (2011) Fast, scalable generation of high-quality protein multiple sequence alignments using Clustal Omega. Mol Syst Biol 7: 539.
