# Supplementary material for: Evolution of p53 Transactivation Specificity through the Lens of a Yeast-Based Functional Assay
Source: PLoS One. 2015 Feb 10;10(2):e0116177. doi: 10.1371/journal.pone.0116177 (PMC4323202; doi:10.1371/journal.pone.0116177)
Supplement: S1 File — Contains Supporting Information on p53 protein sequence evolution. (ZIP) [file pone.0116177.s001.zip › Figures.pdf]

Supplementary figure 1. p53 sequences evolution

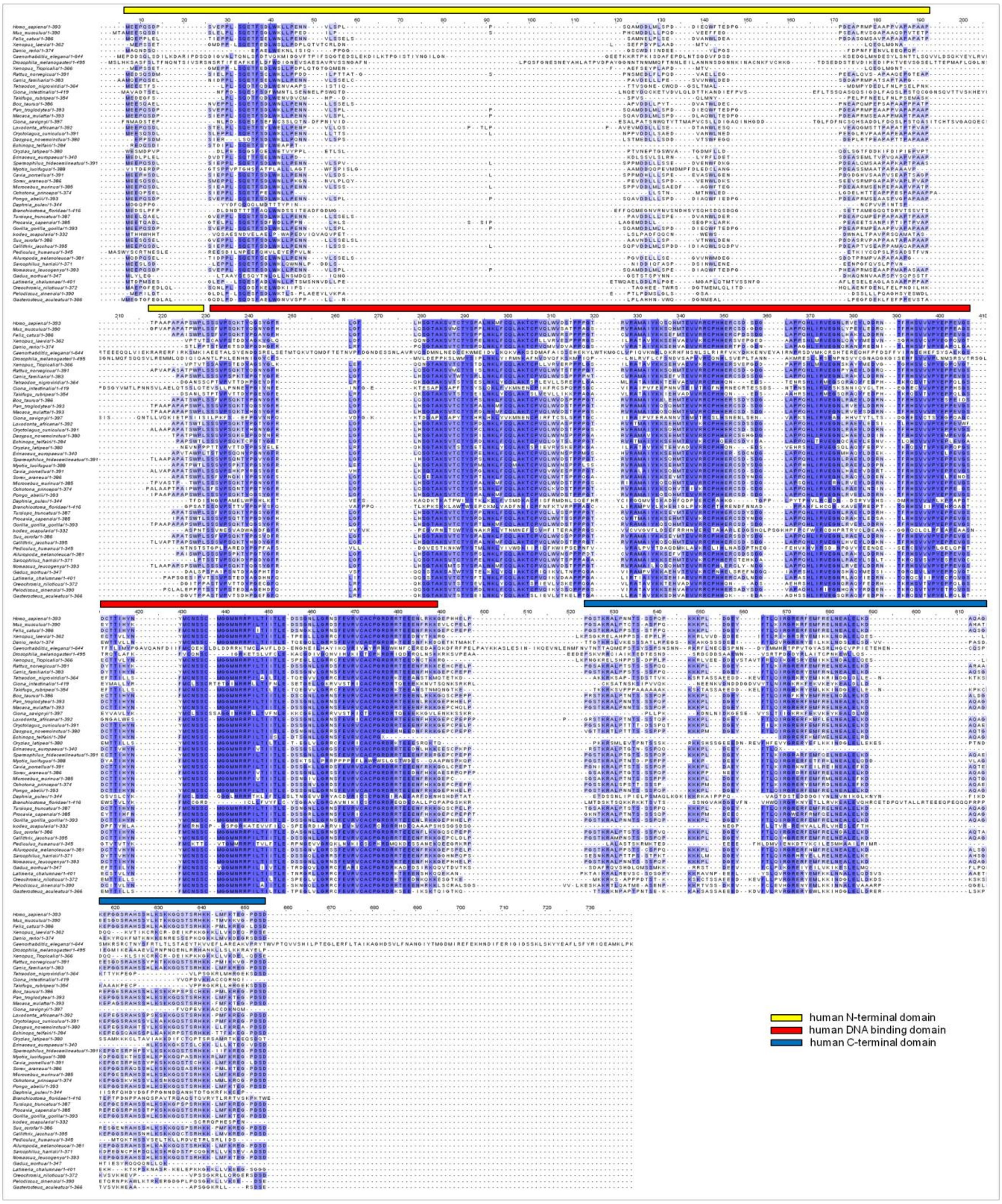

Figure S2

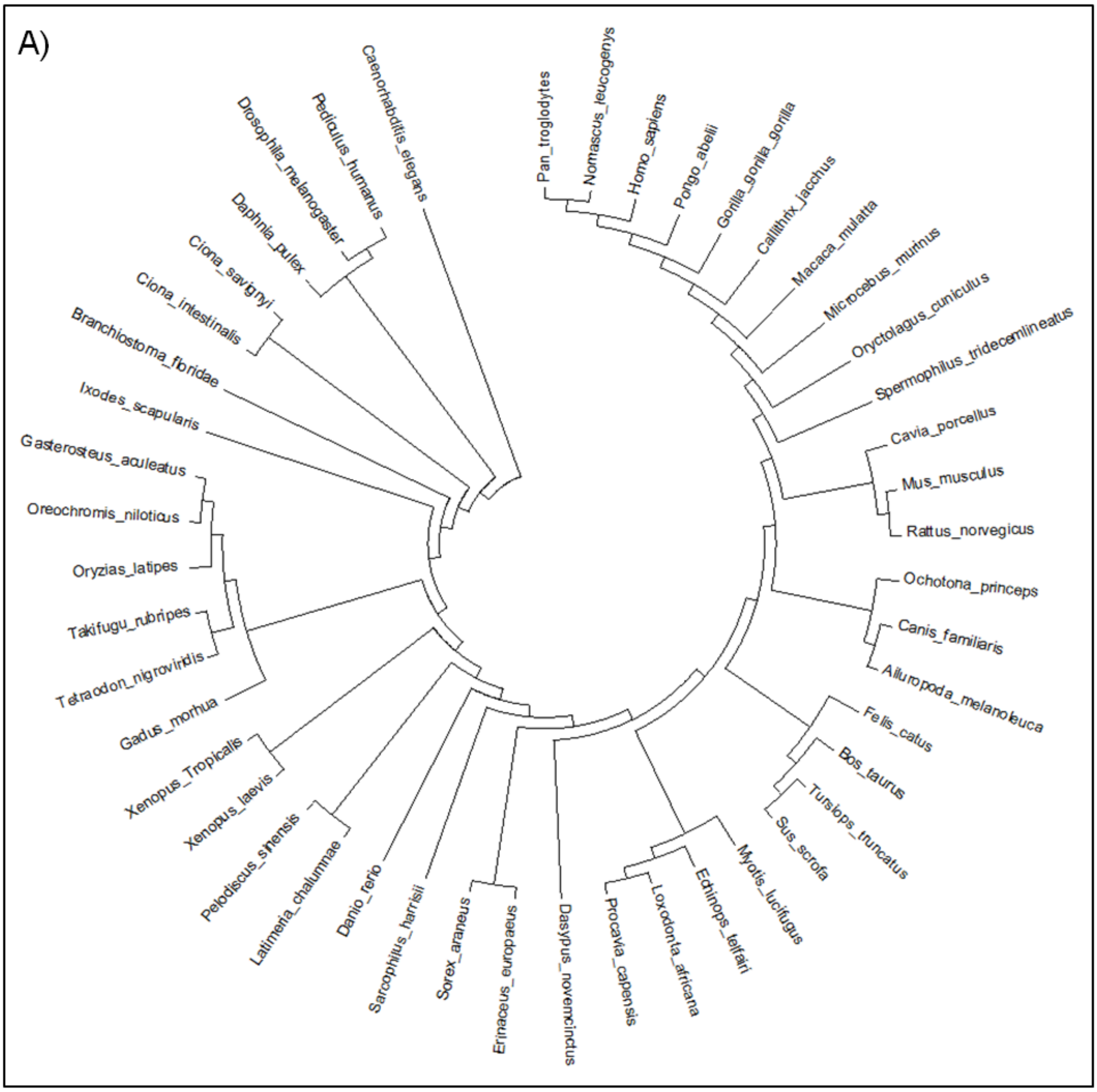

A)
Caenorhabditis_elegans
Pediculus_humanus
Drosophila_melanogaster
Daphnia_pulex
Ciona_savignyi
Ciona_intestinalis
Branchiostoma_floridae
Ixodes_scapularis
Gasterosteus_aculeatus
Oreochromis_niloticus
Oryzias_latipes
Takifugu_rubripes
Tetraodon_nigroviridis
Gadus_morhua
Xenopus_Tropicalis
Xenopus_laevis
Pelodiscus_sinensis
Latimeria_chalumnae
Danio_rerio
Sarcophilus_harrisii
Sorex_araneus
Erinaceus_europaeus
Dasypus_novemcinctus
Procavia_capensis
Loxodonta_africana
Echinops_telfairi
Myotis_lucifugus
Sus_scrofa
Tursiops_truncatus
Bos_taurus
Felis_catus
Ailuropoda_melanoleuca
Canis_familiaris
Ochotona_princeps
Rattus_norvegicus
Mus_musculus
Cavia_porcellus
Spermophilus_tridecemlineatus
Oryctolagus_cuniculus
Microcebus_murinus
Macaca_mulatta
Callithrix_jacchus
Gorilla_gorilla_gorilla
Pongo_abelii
Homo_sapiens
Nomascus_leucogenys
Pan_troglodytes

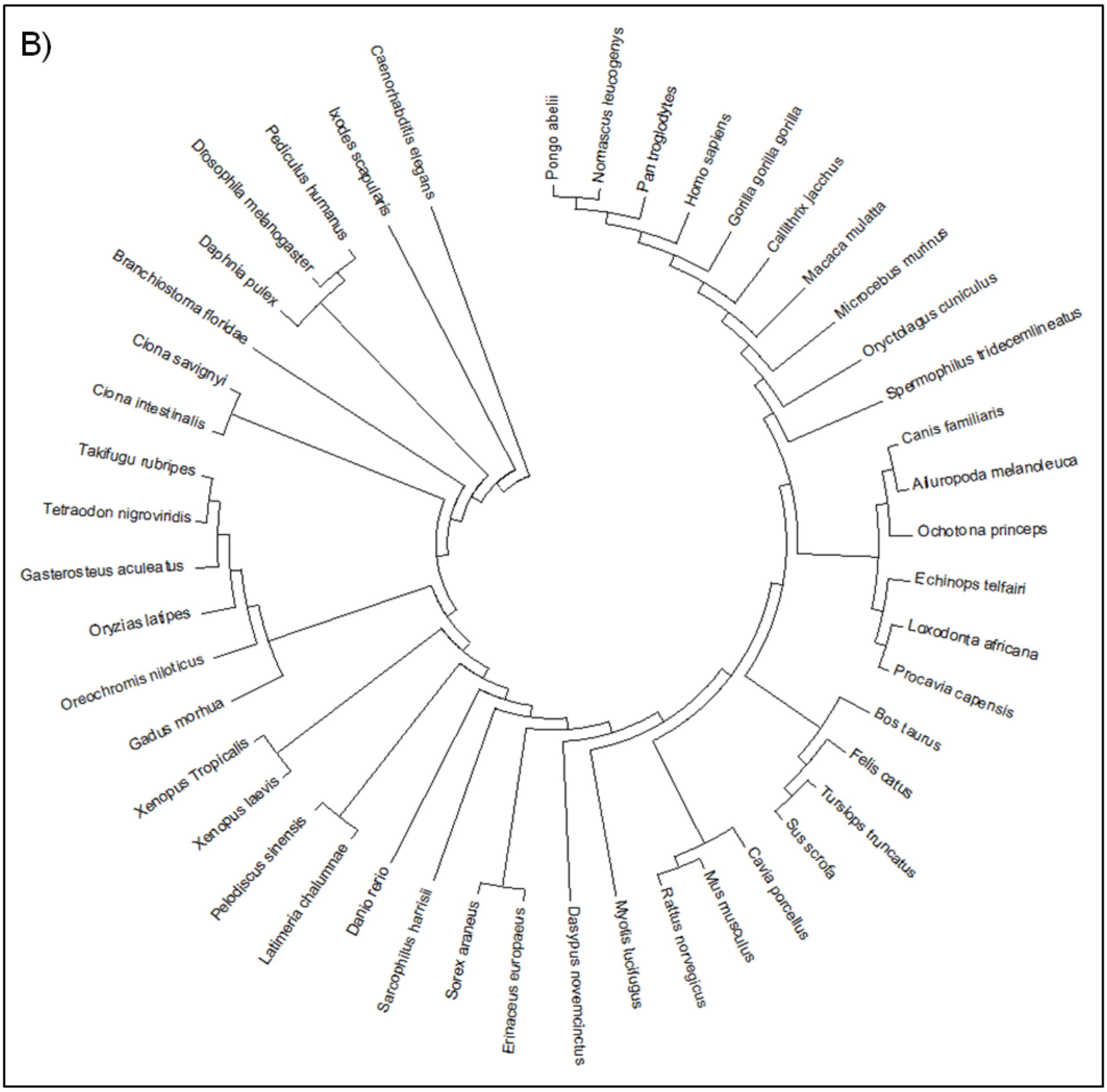
B)
Caenorhabditis elegans
Ixodes scapularis
Pediculus humanus
Drosophila melanogaster
Daphnia pulex
Branchiostoma floridae
Ciona savignyi
Ciona intestinalis
Takifugu rubripes
Tetraodon nigroviridis
Gasterosteus aculeatus
Oryzias latipes
Oreochromis niloticus
Gadus morhua
Xenopus Tropicalis
Xenopus laevis
Pelodiscus sinensis
Latimeria chalumnae
Danio rerio
Sarcophilus harrisii
Sorex araneus
Erinaceus europaeus
Dasypus novemcinctus
Myotis lucifugus
Rattus norvegicus
Mus musculus
Cavia porcellus
Sus scrofa
Tursiops truncatus
Felis catus
Bos taurus
Procavia capensis
Loxodonta africana
Echinops telfairi
Ochotona princeps
Ailuropoda melanoleuca
Canis familiaris
Spermophilus tridecemlineatus
Oryctolagus cuniculus
Microcebus murinus
Macaca mulatta
Callithrix jacchus
Gorilla gorilla gorilla
Homo sapiens
Pan troglodytes
Nomascus leucogenys
Pongo abelii

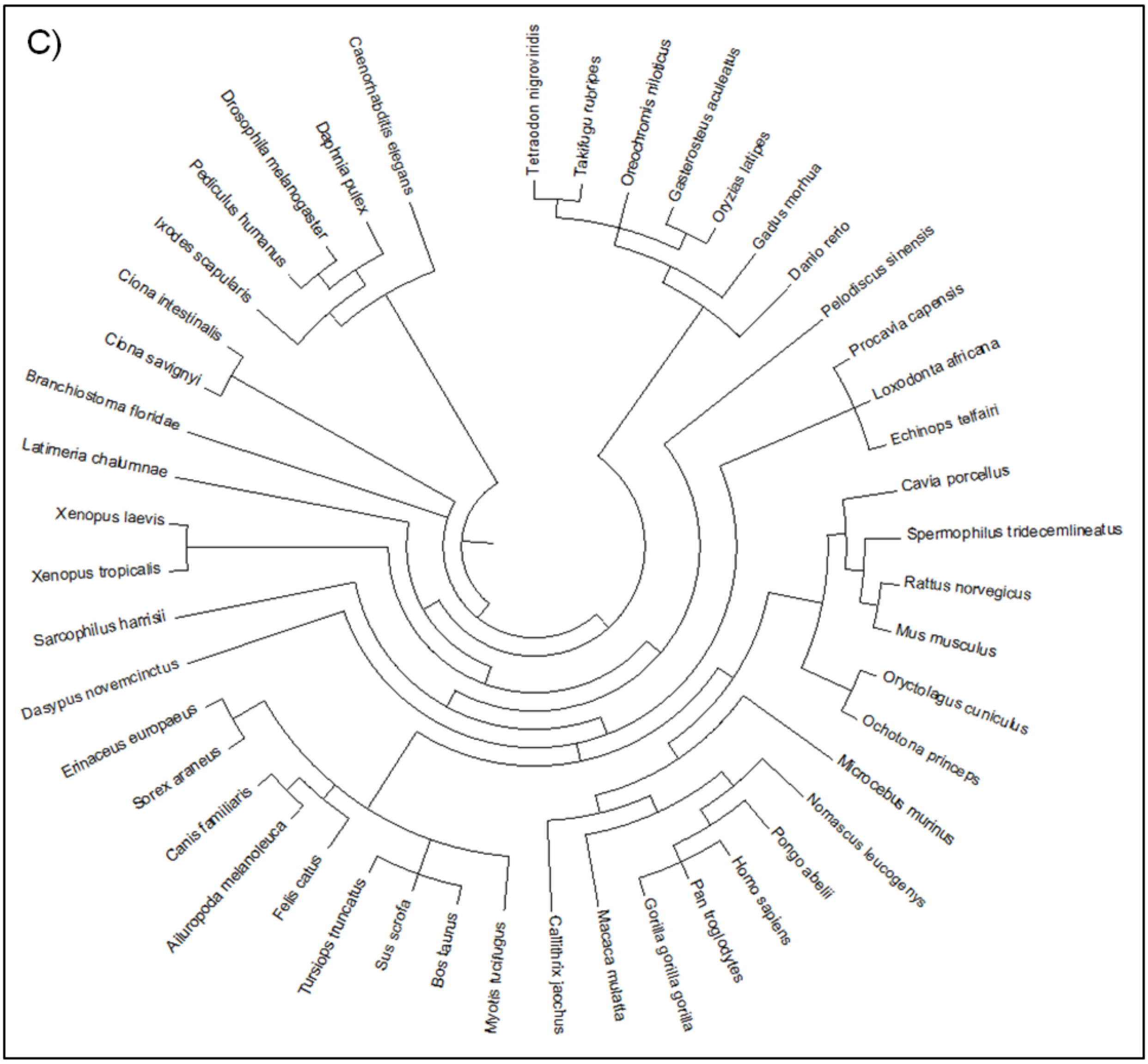
C)
Caenorhabditis elegans
Daphnia pulex
Drosophila melanogaster
Pediculus humanus
Ixodes scapularis
Ciona intestinalis
Ciona savignyi
Branchiostoma floridae
Latimeria chalumnae
Xenopus laevis
Xenopus tropicalis
Sarcophilus harrisii
Dasypus novemcinctus
Erinaceus europaeus
Sorex araneus
Canis familiaris
Ailuropoda melanoleuca
Felis catus
Tursiops truncatus
Sus scrofa
Bos taurus
Myotis lucifugus
Callithrix jacchus
Macaca mulatta
Gorilla gorilla gorilla
Pan troglodytes
Homo sapiens
Pongo abelii
Nomascus leucogenys
Microcebus murinus
Ochotona princeps
Oryctolagus cuniculus
Mus musculus
Rattus norvegicus
Spermophilus tridecemlineatus
Cavia porcellus
Echinops telfairi
Loxodonta africana
Procavia capensis
Pelodiscus sinensis
Danio rerio
Gadus morhua
Oryzias latipes
Gasterosteus aculeatus
Oreochromis niloticus
Takifugu rubripes
Tetraodon nigroviridis

Figure S3

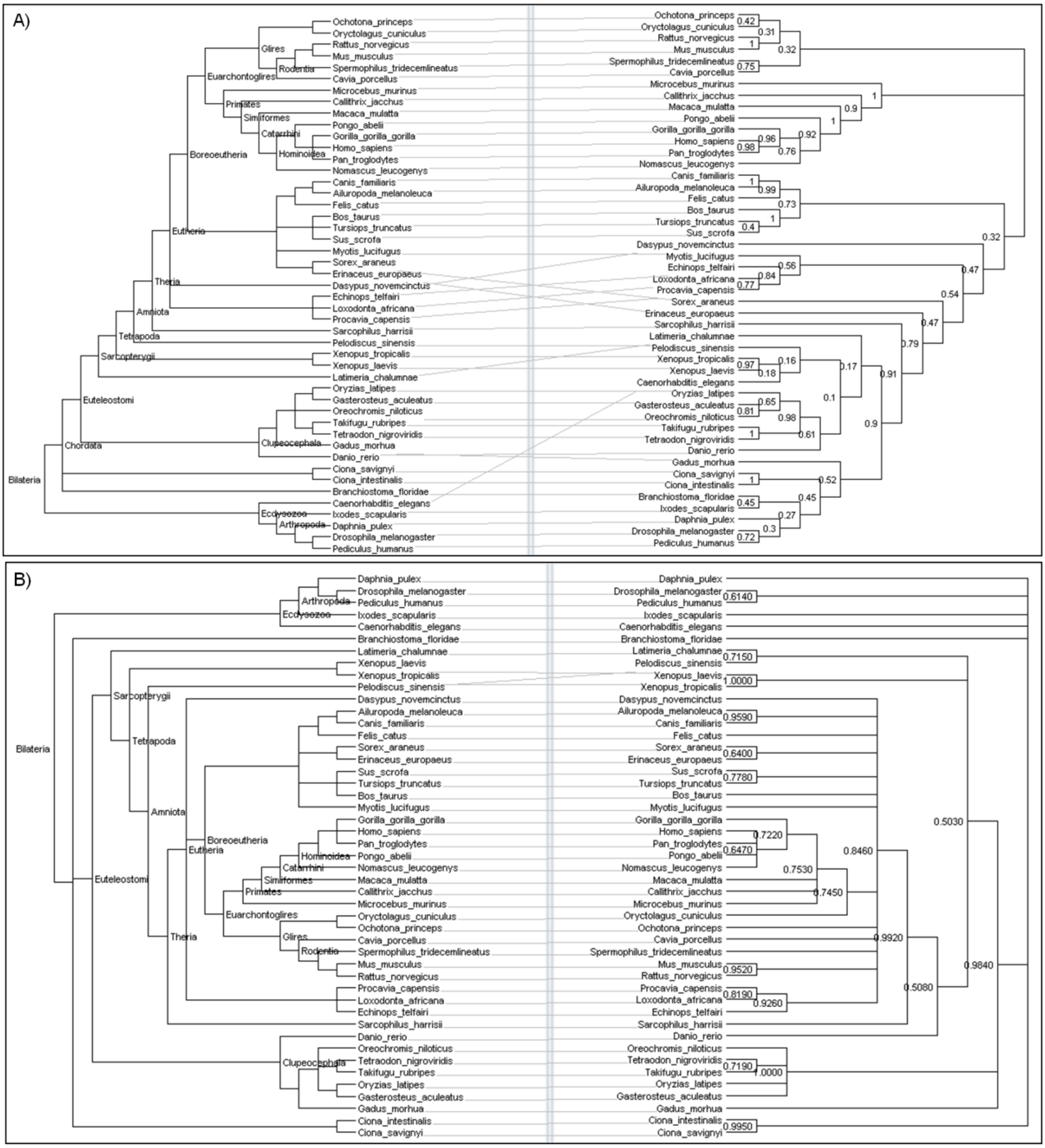

A)
Ochotona_princeps
Oryctolagus_cuniculus
Rattus_norvegicus
Mus_musculus
Spermophilus_tridecemlineatus
Cavia_porcellus
Microcebus_murinus
Callithrix_jacchus
Macaca_mulatta
Pongo_abelii
Gorilla_gorilla_gorilla
Homo_sapiens
Pan_troglodytes
Nomascus_leucogenys
Canis_familiaris
Ailuropoda_melanoleuca
Felis_catus
Bos_taurus
Tursiops_truncatus
Sus_scrofa
Myotis_lucifugus
Sorex_araneus
Erinaceus_europaeus
Dasypus_novemcinctus
Echinops_telfairi
Loxodonta_africana
Procavia_capensis
Sarcophilus_harrisii
Pelodiscus_sinensis
Xenopus_tropicalis
Xenopus_laevis
Latimeria_chalumnae
Oryzias_latipes
Gasterosteus_aculeatus
Oreochromis_niloticus
Takifugu_rubripes
Tetraodon_nigroviridis
Gadus_morhua
Danio_rerio
Ciona_savignyi
Ciona_intestinalis
Branchiostoma_floridae
Caenorhabditis_elegans
Ixodes_scapularis
Daphnia_pulex
Drosophila_melanogaster
Pediculus_humanus
Glires
Rodentia
Euarchontoglires
Primates
Simiiformes
Catarrhini
Hominoidea
Boreoeutheria
Eutheria
Theria
Amniota
Tetrapoda
Sarcopterygii
Euteleostomi
Chordata
Bilateria
Clupeocephala
Ecdysozoa
Arthropoda
B)
Daphnia_pulex
Drosophila_melanogaster
Pediculus_humanus
Ixodes_scapularis
Caenorhabditis_elegans
Branchiostoma_floridae
Latimeria_chalumnae
Xenopus_laevis
Xenopus_tropicalis
Pelodiscus_sinensis
Dasypus_novemcinctus
Ailuropoda_melanoleuca
Canis_familiaris
Felis_catus
Sorex_araneus
Erinaceus_europaeus
Sus_scrofa
Tursiops_truncatus
Bos_taurus
Myotis_lucifugus
Gorilla_gorilla_gorilla
Homo_sapiens
Pan_troglodytes
Pongo_abelii
Nomascus_leucogenys
Macaca_mulatta
Callithrix_jacchus
Microcebus_murinus
Oryctolagus_cuniculus
Ochotona_princeps
Cavia_porcellus
Spermophilus_tridecemlineatus
Mus_musculus
Rattus_norvegicus
Procavia_capensis
Loxodonta_africana
Echinops_telfairi
Sarcophilus_harrisii
Danio_rerio
Oreochromis_niloticus
Tetraodon_nigroviridis
Takifugu_rubripes
Oryzias_latipes
Gasterosteus_aculeatus
Gadus_morhua
Ciona_intestinalis
Ciona_savignyi

Figure S4

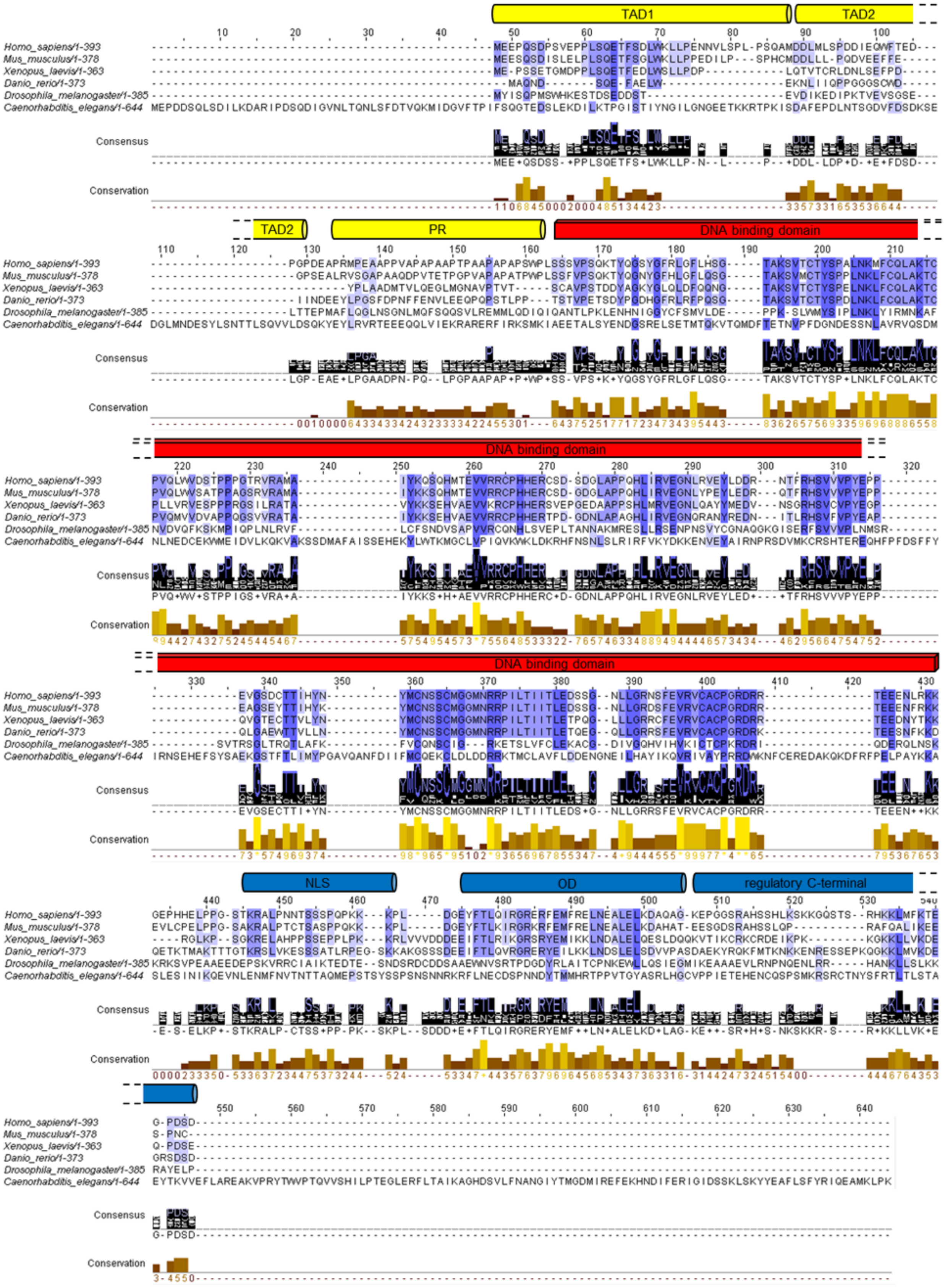

TAD1
TAD2
PR
DNA binding domain
NLS
OD
regulatory C-terminal
Homo_sapiens/1-393
Mus_musculus/1-378
Xenopus_laevis/1-363
Danio_rerio/1-373
Drosophila_melanogaster/1-385
Caenorhabditis_elegans/1-644
Consensus
Conservation

Figure S5

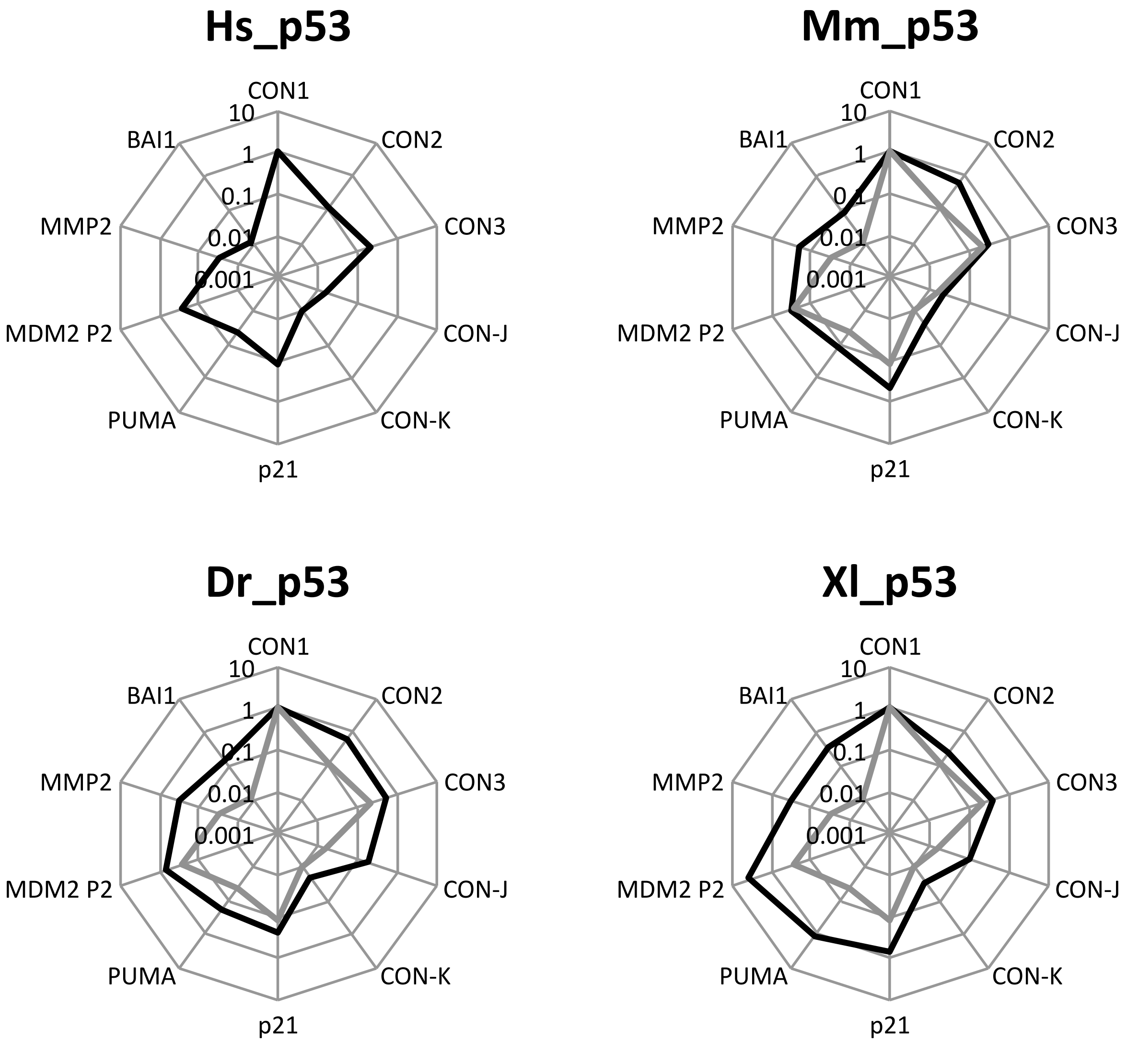

0.008% galactose

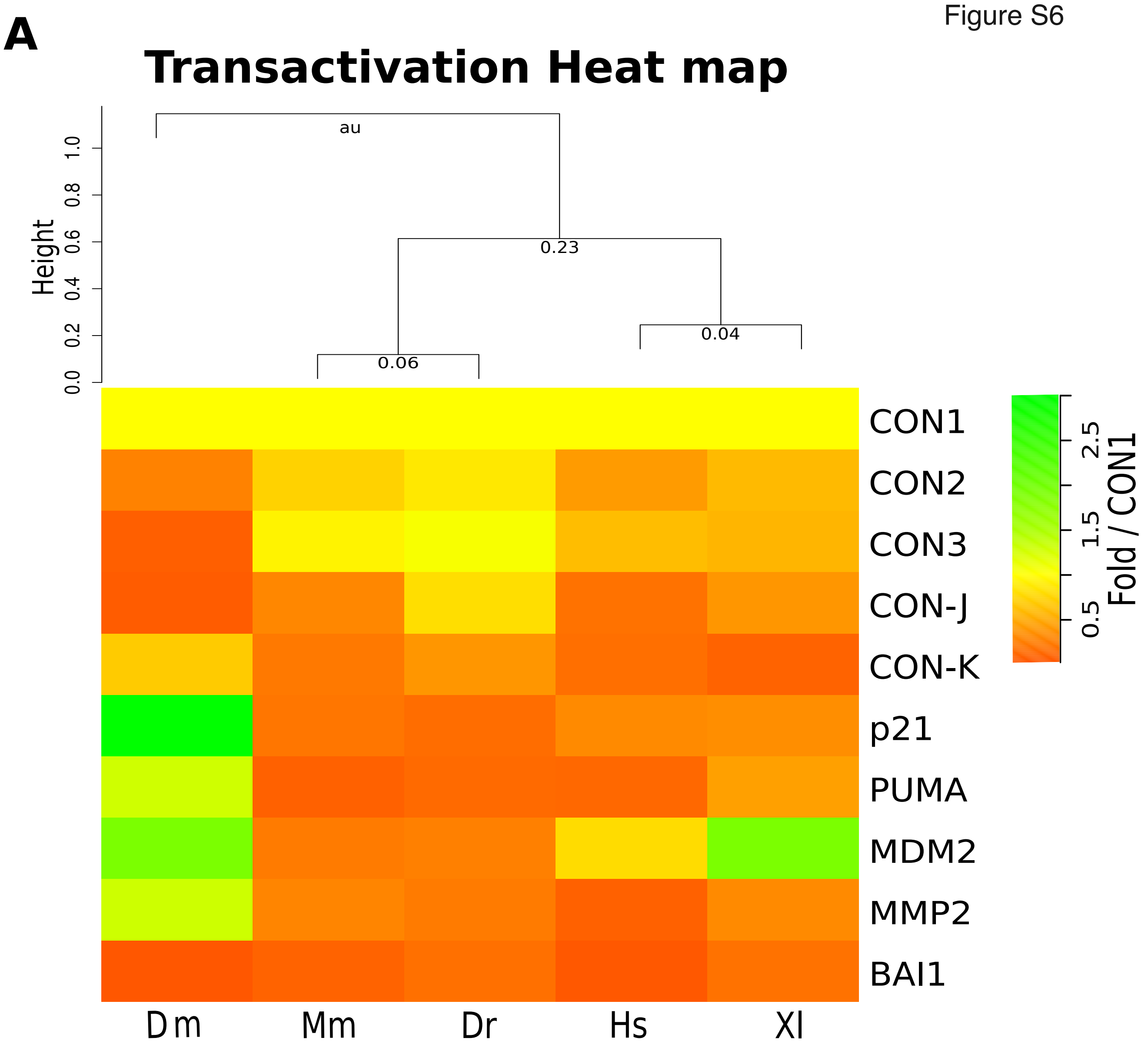
A
Transactivation Heat map
au
0.23
0.06
0.04
Height
0.0
0.2
0.4
0.6
0.8
1.0
CON1
CON2
CON3
CON-J
CON-K
p21
PUMA
MDM2
MMP2
BAI1
Dm
Mm
Dr
Hs
Xl
0.5
1.5
2.5
Fold / CON1

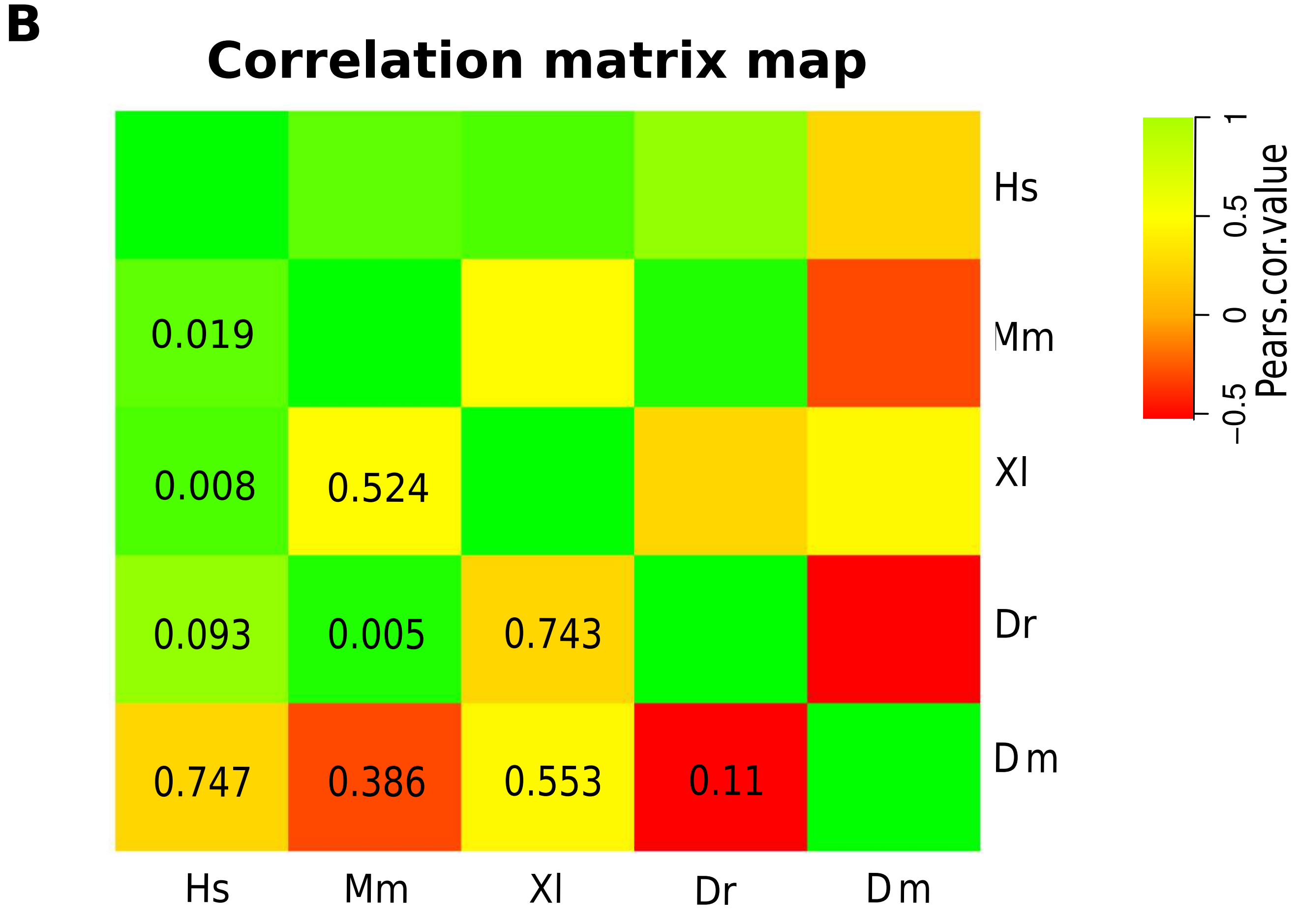
B
Correlation matrix map
0.019
0.008
0.524
0.093
0.005
0.743
0.747
0.386
0.553
0.11
Hs
Mm
Xl
Dr
Dm
−0.5
0
0.5
1
Pears.cor.value

Figure S7

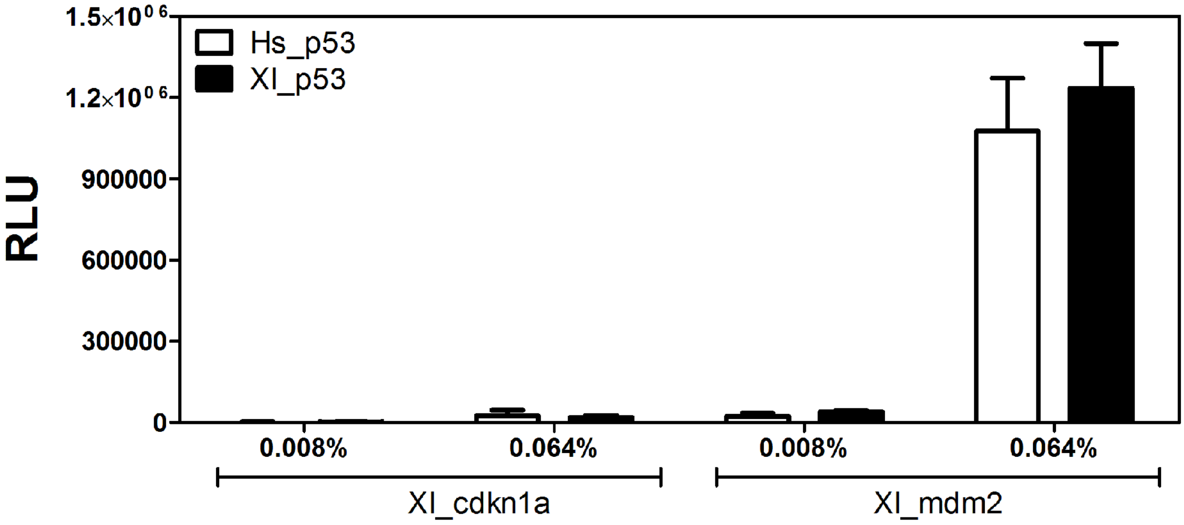

1.5×10⁰ 6
1.2×10⁰ 6
900000
600000
300000
0
RLU
Hs_p53
Xl_p53
0.008%
0.064%
0.008%
0.064%
Xl_cdkn1a
Xl_mdm2
